# Regenerative glutamate release in the hippocampus of Rett syndrome model mice

**DOI:** 10.1371/journal.pone.0202802

**Published:** 2018-09-26

**Authors:** Saju Balakrishnan, Sergej L. Mironov

**Affiliations:** CNMPB (Centre for Nanoscale Microscopy and Molecular Physiology of the Brain, DFG Research Center 103), Institute of Neuro and Sensory Physiology, Georg-August-University, Göttingen, Germany; Indiana University School of Medicine, UNITED STATES

## Abstract

Excess glutamate during intense neuronal activity is not instantly cleared and may accumulate in the extracellular space. This has various long-term consequences such as ectopic signaling, modulation of synaptic efficacy and excitotoxicity; the latter implicated in various neurodevelopmental and neurodegenerative diseases. In this study, the quantitative imaging of glutamate homeostasis of hippocampal slices from methyl-CpG binding protein 2 knock-out (Mecp2^-/y^) mice, a model of Rett syndrome (RTT), revealed unusual repetitive glutamate transients. They appeared in phase with bursts of action potentials in the CA1 neurons. Both glutamate transients and bursting activity were suppressed by the blockade of sodium, AMPA and voltage-gated calcium channels (T- and R-type), and enhanced after the inhibition of HCN channels. HCN and calcium channels in RTT and wild-type (WT) CA1 neurons displayed different voltage-dependencies and kinetics. Both channels modulated postsynaptic integration and modified the pattern of glutamate spikes in the RTT hippocampus. Spontaneous glutamate transients were much less abundant in the WT preparations, and, when observed, had smaller amplitude and frequency. The basal ambient glutamate levels in RTT were higher and transient glutamate increases (spontaneous and evoked by stimulation of Schaffer collaterals) decayed slower. Both features indicate less efficient glutamate uptake in RTT. To explain the generation of repetitive glutamate spikes, we designed a novel model of glutamate-induced glutamate release. The simulations correctly predicted the patterns of spontaneous glutamate spikes observed under different experimental conditions. We propose that pervasive spontaneous glutamate release is a hallmark of Mecp2^-/y^ hippocampus, stemming from and modulating the hyperexcitability of neurons.

## Introduction

Around 80% of neurons in the CNS use glutamate as transmitter, but it also has a plethora of other physiological and pathological actions. The pathological pathways are thought to originate after the escape of glutamate from the synaptic cleft. Excess glutamate is normally removed by transporters in the astrocytes and neurons, but their capacity is limited [1–3]. The clearance mechanisms may become inefficient during intense neuronal activity that can lead to a persistent increase in the ambient glutamate. Sustained glutamate levels were proposed to mediate the aetiology of various neurological diseases and psychiatric disorders. For example, the excitotoxic actions of glutamate are involved in neurodegenerative diseases such as amyotrophic lateral sclerosis [4], Huntington’s [5, 6] and Alzheimer’s diseases [7]; and brain insults [8]. Aberrant glutamate handling is also postulated as an immediate cause of epilepsy [9], schizophrenia [10], and spreading depression [11].

**Fig 1:**
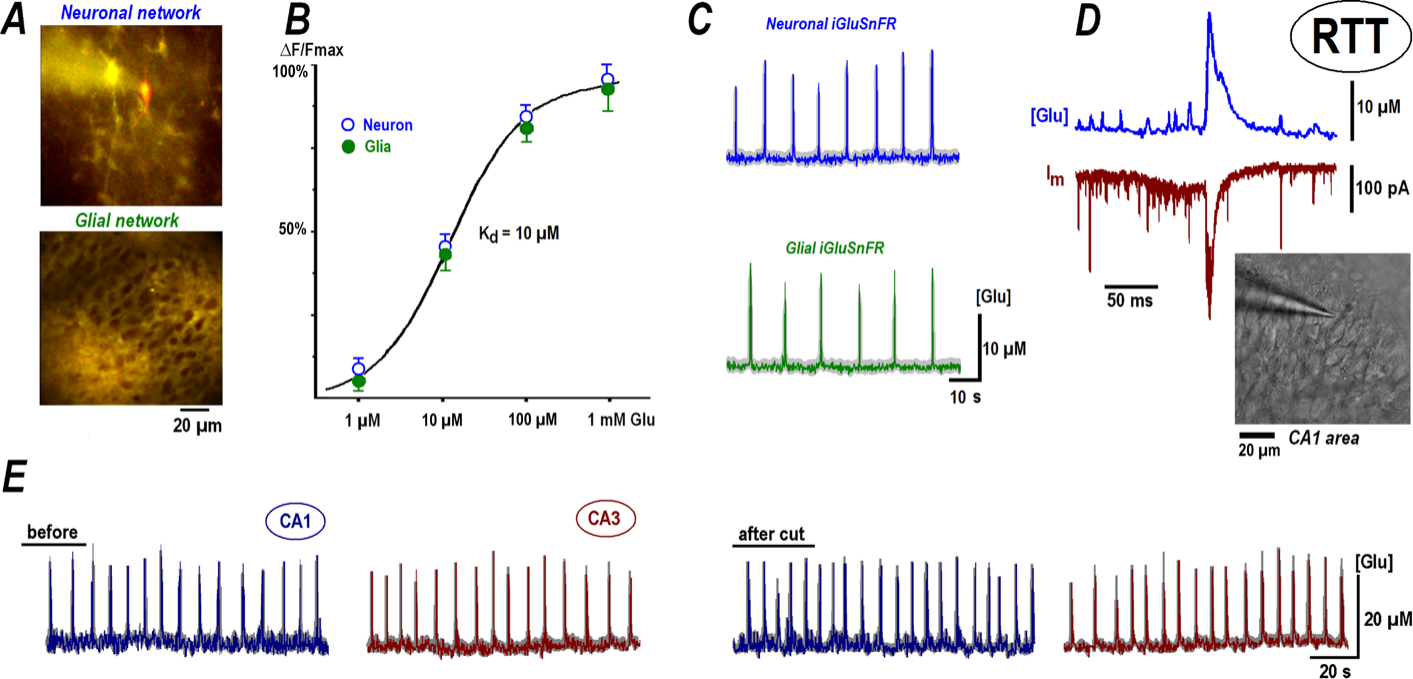
Glutamate imaging reveals spontaneous glutamate spikes in CA1 hippocampus. ***A***–Representative fluorescence images of hippocampal slices transduced with iGluSnFR sensor (encapsulated into AAV5 vectors) targeted to neurons and astrocytes as indicated. ***B***–The dose-response curves for sensor responses in neurons and astrocytes were fitted well by the Michaelis-Menten-like equation(Δ*F*/*Fmax*) = [*Glu*]/(*Kd* + [*Glu*]). The concentration dependencies coincided in both cell types and corresponded to the same *K*_*d*_ = 10 μM. Mean data for each concentration is derived from four individual experiments. ***C–***Sample traces of repetitive glutamate releases recorded with neuronal (*top*) and glial sensors (*bottom*) in CA1 area in slices from RTT animals (see also Fig 2). Thin traces represent mean changes averaged over 12 cells and thick grey backgrounds show ± SEM. ***D–***Correlation between local glutamate spikes (*top trace*) and excitatory postsynaptic synaptic currents (EPSC, lower trace) measured in CA1 neurons at the holding potential of -70 mV. Negative deflections in whole-cell recording indicate EPSCs that eventually produced a ‘synaptic drive’; a correlate of the burst of action potentials in the current-clamp mode. Note the good temporal correspondence between EPSCs and glutamate transients. The inset shows DIC-image with patched CA1 cell. ***E–***Spontaneous activity in CA1 and CA3 areas. The traces present spontaneous glutamate transients in naïve slices from RTT animals before and after mechanical separation of CA1 and CA3 areas.

Despite its crucial functional significance, and after a multitude of biochemical studies, we do not yet know in detail how the ambient glutamate levels are established, maintained and regulated within the brain. Until recently there was no reliable tool for measuring glutamate levels within neuronal tissue with a sufficient temporal and spatial resolution. Previously used techniques such as glutamate-sensing microprobes [11] can only provide a temporal and spatial resolution of >1 s and >100 μm, respectively. But the glutamate changes in the brain can be much faster and finer. The design of a genetically encoded fluorescent glutamate sensor, iGluSnFR [12] is a quantum leap in researching glutamate homeostasis in situ. This sensor is coupled to human synapsin or GFAP (glial fibrillary acidic protein) promoters, which drive its exclusive expression in the neurons or astrocytes, respectively. The targeted sensor is localized to respective plasma membranes and senses changes in extracellular glutamate concentrations from 0.1 μM to 1 mM, with spatial and temporal resolutions of ~1 μm to ~1 ms, respectively ([12], see also Fig 1).

In this study we used iGluSnFr to assess glutamate homeostasis in the hippocampus of a mouse model of Rett Syndrome (RTT). This neurodevelopmental disorder stems from improper maturation of the synapses due to the loss of function of the methyl-CpG binding protein 2, Mecp2 [13]. The hallmark of RTT is the hyperexcitability of neurons that is assumed to make the networks vulnerable to epileptic seizures [14, 15]. Hyperexcitability in RTT is thought to stem from imbalances between excitation and inhibition [16], which can be reversed by the NMDA receptor antagonist ketamine [17]. Several previous reports indicate an abnormal glutamate handling in RTT. Okabe et al. [18] reported a diminished glutamate clearance in Mecp2-deficient astrocytes in culture. Maezawa and Jin [19] showed the toxic effects of glutamate production by Mecp2-null microglia. EEG and *in vivo* glutamate measurements demonstrate severe sleep dysfunction in RTT mice that were related to abnormal glutamate changes in the frontal cortices [20].

Using glutamate imaging with iGluSnFR, in the hippocampus of Mecp2^-/y^ mice, we report that the ambient glutamate levels are significantly higher than in the wild-type (WT). In RTT we unexpectedly observed a novel phenomenon of repetitive glutamate transients. They appeared synchronously in the imaged area and were generated in phase with the bursts of the action potentials (AP) in the CA1 neurons. In WT slices such transients were rare and had smaller amplitudes and frequencies, when observed. The pattern of glutamate transients was modified after modulation of glutamate uptake, release (evoked and spontaneous) and diffusion. This pharmacological evidence posits presynaptic endings as an immediate source of the regenerative glutamate release.

Repetitive glutamate spikes seen in the RTT hippocampus critically depend on the activity of hyperpolarization-activated (HCN), and voltage-sensitive calcium channels (VSCC). HCN and VSCC channels in WT and RTT CA1 neurons showed different voltage dependencies that have not been reported before. The concerted actions of AMPA receptors, glutamate transporters, HCN and T- and R-type calcium channels orchestrate the generation of regenerative glutamate transients in the RTT hippocampus. Such cross-talks can provide prerequisites for hyperexcitability in RTT, inducing seizures and other RTT related pathological features. We propose that chronically elevated glutamate established due to a less effective glutamate uptake in the hippocampus could be one of the major factors that contribute to the onset and development of RTT phenotype.

## Materials and methods

### Preparation

All animals were housed, cared for and euthanized in accordance with the recommendations of the European Commission (No. L358, ISSN 0378–6978), and protocols were approved by the Committee for Animal Research, Göttingen University. Experiments were performed using the mouse model for Rett syndrome; strain B6.129P2(C)-Mecp2tm1-1Bird [21]. The mice were obtained from the Jackson Laboratory (Bar Harbor, ME, USA) and maintained on a C57BL/6J background. Hemizygous mutant Mecp2^-/y^ males (knock-outs) were generated by crossing heterozygous Mecp2^+/−^ females with C57BL/6JWT males. All mice were routinely genotyped in accordance with the Jackson Laboratory genotyping protocols that unequivocally distinguished between the wild-type and knock-out mice.

Organotypic slices were prepared using the procedure described in [22] with modifications as described [15, 23]. In summary, at postnatal day P3 the animals were anaesthetized with isoflurane and decapitated. Both hippocampi were carefully isolated and 12 to 18 transverse 250 μm thick slices were cut and placed on support membranes (Millicell-CM Inserts, PICMORG50; Millipore). The surface of the slice was continuously exposed to the incubator gas mixture. The medium (50% MEM with Earle's salts, 25 mM HEPES, 6.5 mg/ml glucose, 25% horse serum, 25% Hanks solution buffered with 5 mM Tris and 4 mM NaHCO_3_, pH 7.3) was changed every second day. The general chemicals were from Sigma (Deisenhofer, Germany). The agonists and antagonists of the ion channels and receptors were from Tocris (Bristol, UK) and Alomone Labs (Jerusalem, Israel). The stock solutions were made either in DMSO or in ACSF. Fluo-4, fura-2 AM and Alexa 568 were purchased from Thermo Fisher (Germany). Slices were transduced using an adeno-associated virus vector carrying glutamate sensor [12] targeted to neurons (AAV5.hSyn.iGluSnFr.WPRE.SV40) or astrocytes (AAV5.GFAP.iGluSnFr.WPRE.SV40). The constructs were purchased from Penn Vector Core (Department of Pathology and Laboratory Medicine; U. Pennsylvania) and the organotypic slices were transduced with them two days after plating. The experiments were performed P10 onwards, after distribution of the sensor was uniform throughout the cells in the slices. As in our previous studies using genetically encoded sensors [23–25], we did not notice any modifications in the morphological and electrophysiological characteristics of the transduced neurons in comparison with naive neurons.

During the experiments, the membrane with the attached slice was fixed on a coverslip in the recording chamber and continuously superfused at 34 ^o^C with artificial cerebrospinal fluid (ACSF) containing: 138 mM NaCl, 3 mM KCl, 1.5 mM CaCl_2_, 1 mM MgCl_2_, 30 mM HEPES, 1 mM NaH_2_PO_4_, 10 mM glucose, at pH 7.4. The volume of the perfusion chamber was maintained at approximately 2 ml with a flow rate of 10 ml/min. Solutions were exchanged by replacing a distal reservoir with another one containing drugs, and new solutions reached the chamber within 30 s.

### Imaging

The optical recording system included an upright microscope (BX51, Olympus, Hamburg, Germany) equipped with a monochromatic light source (CAIRN, UK). The cells were viewed under a 40× objective (LUMplanFI, N. A. 0.8). Images from a cooled CCD camera (Andor Ixon, Belfast, UK) were digitized (256×256 pixels at 12 bit resolution) and collected with ANDOR software. For measuring the glutamate increase with a faster temporal resolution an iXon Ultra 888 camera (Andor, Belfast, UK) was used. With this camera, when the image was cropped to 80x80 pixels, we achieved an acquisition time ranging from 0.5 to 2 ms. The images were analyzed offline with Metamorph software (Princeton Instruments).

Glutamate imaging was done using 470/525±10 nm (49002, Chroma technology, Olching, Germany) excitation/emission wavelengths. In a set of experiments, we imaged intracellular calcium using either bulk loading of slices with fura-2 AM (5 μM for 30 min at 37 ^o^C) or intracellular dialysis of the patched cell with fluo-4 (100 μM). The excitation/emission wavelengths for fura-2 were 380/525±10 nm (79001, Chroma technology, Olching, Germany). Fluo-4 was excited at 470 nm, and the emission was collected at 525±10 nm (49002, Chroma technology, Olching, Germany). To visualize neuron morphology, Alexa 568 (100 μM) was added to patch pipette solution. The dye was excited at 565 nm and excitation/emission light was separated with dichroic mirror centered on 585 nm (T585lpxr, Chroma technology, Olching, Germany), and emission was collected through 610/75 nm filter (45186, Chroma technology, Olching, Germany).

Glutamate levels were calculated from the measured relative increase in fluorescence *ΔF/F*_*o*_ using the inverted form of the Michaelis-Menten-like equation, Δ*F*/*Fo* = *Fmax* [*Glu*]/(*Kd* + [*Glu*]) where *F*_*o*_ is the resting (background-subtracted) fluorescence level, and the values of *F*_*max*_ and *K*_*d*_ were derived from the calibrations (see Results and Fig 1B). The calcium levels were obtained from fluo-4 signals using the same formula with [*Ca*] instead of [*Glu*] and *K*_*d*_ = 0.35 μM [26]. In fura-2 imaging, the calcium levels were calculated from measurements with 380 nm excitation using the equation *Fo*/Δ*F* = *Fmax*/(*Kd* + [*Ca*]) with *K*_*d* =_ 0.24 μM [27]. Single wavelength calcium measurements were validated using dual excitation of fura-2 at 360 (isosbestic point) and 380 nm.

### Electrophysiology

Patch electrodes were pulled from borosilicate glass (WPI, Berlin, Germany) and had resistances of 2–3 MΩ when filled with the solution containing 110 mM K^+^-gluconate, 5 mM KCl, 50 mM HEPES, 0.005 mM EGTA, 4 mM MgSO_4_, 4 mM ATP, 0.2 mM GTP, 9 mM phosphocreatine, at pH 7.4. For HCN and calcium current recordings, we used a Cs^+^ based solution containing 92 mM CsMeSO_4_, 43 mM CsCl, 5 mM TEA-Cl, 0.4 mM EGTA, 1 mM MgCl_2_, 10 mM HEPES, 4 mM ATP, 0.4 mM GTP, at pH 7.4. Patch-clamp signals were recorded with an EPC-9 (HEKA, Germany) amplifier as described previously [28]. Membrane currents were filtered at 3 kHz (−3 dB), digitized at 10 kHz, and stored for off-line analysis. Schaffer collateral inputs to CA1 neurons were stimulated using extracellular Teflon-coated platinum electrodes with currents pulses from 10 to 100 μA. Analysis of spontaneous and miniature excitatory synaptic currents (sEPSCs and mEPSCs, respectively) was made as described previously [29]. Shortly after exclusion of episodes of bursting activity, the first two moments *mn* = ∫ *ln* (*t*) *dt* of the current *I*(*t*) was calculated. Mean amplitudes (*A*) and frequencies (*f*) of EPSCs were obtained as, *A* = 4*m*2*τ*/*m*1 and *f* = *m*2/4*τm*1, where *τ* is the decay constant of synaptic current determined from its mean waveform.

### Model of glutamate-induced glutamate release (GIGR)

We modeled a network of densely packed glutamate release sites (synapses) in 200x200 μm square area. The time-dependent glutamate changes in this model are described according to the reaction-diffusion (RD) equation

d[Glu]/∂t=DΔ[Glu]–Uptake+Release (1)

The first term in the right-hand part *DΔ*[*Glu*] represents glutamate diffusion (*Δ* is the Laplacian and *D* = 0.3 μm^2^/ms is the diffusion coefficient of glutamate, [30]). The ‘uptake’ is described by the Michaelis-Menten equation

Uptake=Vmax[T][Glu]/(Kd+[Glu]) (2)

Where [*T*] = 1 mM is the concentration of glutamate transporters, *V*_*max*_ = 0.1 s^-1^ is the maximal uptake by transporters and *K*_*d*_ = 10 μM stands for their affinity to glutamate. Glutamate release was presented as a sum of synchronous and spontaneous events. The rate of spontaneous release was set to 5 Hz, in accordance with the experimental data. A synchronous release by the product was presented with a maximal rate *R* and the two variables *A* and *I* that describe activation and inactivation (desensitization) of GIGR mechanism. Both activatory and inhibitory gates depend on ambient glutamate level. The latter one terminates a release event and determines the time required for the release machinery to recover and enter a new activation cycle, which establishes the repetitive activity. A minimal cyclic scheme is

$$C-\frac{a}{}\to A-\frac{b}{}\to I-\frac{d}{}\to C$$ (3)

The number of parameters was set to a minimum and the inclusion of backward reactions does not influence the model behavior. The first rate constant from closed (*C*) to active (*A*) state was *a* = 2.108 *M* − 1 *s* − 1, close to the diffusional on-rate constant and *k*_*on*_ value for glutamate transporters [31, 32]. Other constants, *b* = 100 s^-1^ and *d* = 10 s^-1^, were chosen to reproduce experimental data (see Figs 3 and 4 below). Changes in the value of *b* did not modify the results of simulations. A modification of the value of *d* altered the interval between glutamate spikes as it sets a time for the release mechanism to recover from inactivation. The time-dependent changes in activation and inactivation variables at each release site obey the ordinary differential equations (ODE)

dA/dt=aC–bA

dI/dt=−dI+bA (4)

where *A*, *C* and *I* stand for the occupancies of activated, closed and inactivated states, respectively. Glutamate release sites are placed 1 μm apart and the maximal release rate is set to *R* = 1 mM/ms, in accordance with the data for CA1 area [30]. For each site, the corresponding single ODEs (4) were solved by the Euler backward method and RD Eq (1) was integrated by the Lax algorithm. The time and space steps were 0.01 s and 0.1 μm, respectively. The code for the model was programmed on Turbo-Pascal. The performance of the program was stable and calculated solutions did not depend on the time and space steps.

### Statistics

Approximately equal numbers of neurons were measured in parallel from wild-type and Mecp2-null mice. Each test in this study was repeated with at least four different preparations. The mean data in imaging was routinely obtained by analyzing the simultaneous responses of >12 cells in the image field. For each of the experiment sets, ± SEM were compared using Student's *t* test, with *P*<0.05 being the criterion for statistical significance. The results of independent experiments (WT vs RTT) were compared with the Mann-Whitney-*U*-test, considering *P*<0.05 as statistical significance. The traces representing imaging experiments in the Figures show mean responses overlaid upon thick grey backgrounds indicating variation between traces from individual experiments. All data generated or analyzed during this study is included in this article and in the Supporting information).

## Results

The expression of glutamate sensors, tagged to synapsin or GFAP, showed distributions that matched morphological features of the corresponding neuronal or glial networks, respectively (Fig 1A). We first calibrated the glutamate signals reported by these sensors. The measurements were done in the presence of tetrodotoxin (TTX, 100 nM) to block active responses, and glutamate uptake was inhibited by 10 μM DL-threo-beta-benzyloxyaspartate (TBOA, 50 μM). Stepwise elevation of bath glutamate levels established new fluorescence levels within 2 min. The dose-response curves of neuronal or glial sensors were identical (Fig 1B) and well described by the Michaelis-Menten equation with a dissociation constant of ~10 μM, close to that reported previously in cell culture [12].

### Synchronous glutamate spikes as a hallmark of RTT

In naïve (untreated) hippocampal organotypic slices from Mecp2^-/y^ mice, the ambient glutamate levels fluctuated and showed brief increases up to 20 μM that appeared regularly every 10 to 20 s. Neuronal and glial sensors reported similar glutamate signals (Fig 1C). This close correspondence by both sensors can be explained by the fact that the membrane-targeted glutamate sensors are exposed to the same extracellular milieu (30–100 nm wide) bordered by neurons and glia. The distances <200 nm are beyond the spatial resolution of custom fluorescence microscopy set-ups. The close apposition of glial and neuronal sensors to release sites thus precludes the acquisition of signals generated exclusively in neurons or astrocytes. The data obtained with the two sensors were therefore pooled together for subsequent analysis.

Fig 1D shows a simultaneous recording of glutamate transients and spontaneous excitatory synaptic currents (sEPSCs) in the voltage-clamp mode. Synchronous glutamate release closely matched synaptic activity. Most of the measurements in this study were made from the CA1 area of the organotypic slices with simultaneous patch-clamp recording. Hyperactive CA3 pyramidal neurons have been previously proposed to drive excessive activity in other areas of the hippocampus, which may culminate in limbic seizures. The voltage imaging of acute hippocampal slices from RTT animals [33] shows hyperexcitability in both CA1 and CA3 which was absent in isolated CA1 minislices. Our patch-clamp recordings confirm hyperexcitability in the CA1 neurons in organotypic and acute slices from RTT animals (S1 Fig and S1 File). In the naïve RTT slices mean amplitudes of spontaneous glutamate transients were 16.43 ± 3.02 μM in CA1 and 15.67 ± 3.72 μM in the CA3 area (Fig 1E). These transients persisted after the mechanical separation of CA1 (16.90 ± 2.97 μM) and CA3 areas (14.64 ± 4.13 μM, n = 10 slices). Spontaneous activity can hence be considered as autonomous in each region and may utilize similar signaling mechanism(s) that we examined further (see below).

**Fig 2:**
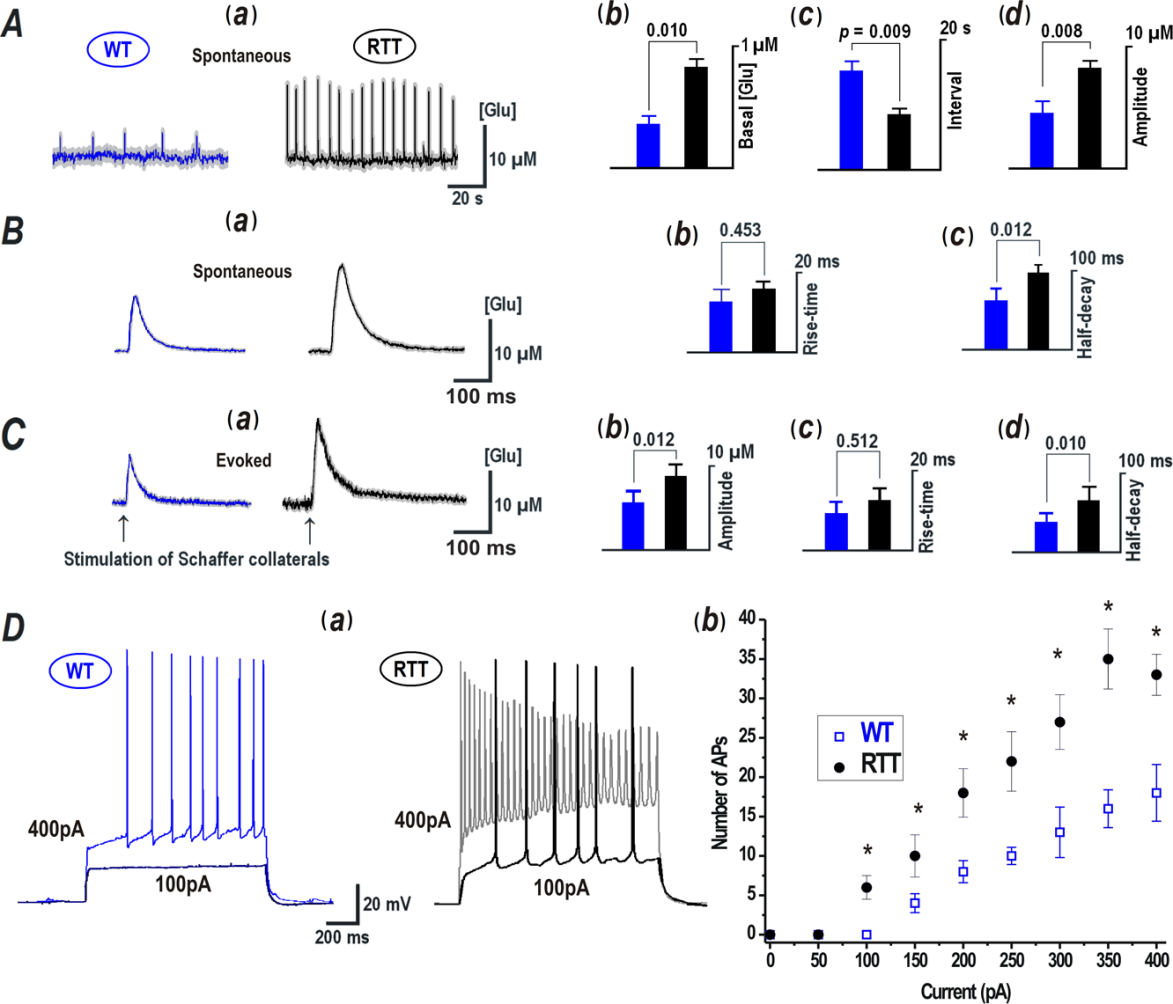
Glutamate transients and excitability of CA1 neurons. ***A***–(***a***) Glutamate transients in the slices from wild-type (WT) and Mecp2 null (RTT) mice, presented as average traces (obtained from 12 neurons in the image field of a single representative experiment) and overlaid upon grey background (± SEM). The traces and mean data obtained from WT and RTT slices here and below are presented in different colors. The histograms on the right present basal glutamate levels (***b***), mean intervals between the spikes (***c***), and their amplitudes (***d***). The data was obtained from 24 ‘rhythmic’ WT slices (blue bars) out of the 158 examined and from 108 RTT slices out of the 142 examined (black bars). ***B*–(*a*)** Representative spontaneous glutamate transients in expanded scale. Note the bigger and slower glutamate transients in RTT. (***b***) Cumulative comparison of rise times measured as 10 to 90% increase. (***c***) Decay times of spontaneous glutamate transients. ***C***—(***a***) Glutamate changes evoked by stimulation of Schaffer collaterals at the times indicated by the arrows under the traces. The transients in WT and RTT showed similar rise-times (***b***) but significantly different decay times (***c***). Statistical data are means ± SEM of six independent experiments. All the above data is evaluated using Mann-Whitney-*U*-test. Corresponding *P* values are given in histograms. All differences were significant, except the rise-times. ***D*—**Enhanced excitability in RTT. (***a)*** Shown are the responses of CA1 neurons of WT and RTT to the two current injections (100 and 400 pA,). The input-output relationships are shown on the right (***b***). Mean data was obtained from 12 cells examined in six different preparations from WT and RTT animals.

The repetitive glutamate transients were observed much more frequently in slices from RTT animals than in WT. The transients occurred spontaneously in 108 out of 142 naïve RTT slices, and only in 24 out of 158 WT slices investigated (Fig 2Aa). Basal glutamate levels are, in general, determined by the opposite processes of glutamate release, that is, by uptake from, extracellular space (Eq (1) in Methods). A faster uptake produce lower ambient glutamate: the basal levels were 0.43 ± 0.03 μM in WT (n = 158 slices) and 0.90 ± 0.04 μM in RTT (n = 142 slices), respectively (Fig 2Ab, *P*<0.05, Mann-Whitney-*U*-test). The amplitudes and frequencies of spontaneous glutamate transients in RTT slices (15.91 ± 3.54 μM and 0.22 ± 0.04 Hz) were significantly bigger that in WT (6.13 ± 1.23 μM and 0.10 ± 0.03 Hz, *P*<0.05, Mann-Whitney-*U*-test, Fig 2A). Individual transients in WT and RTT slices also had different waveforms (Fig 2Ba). Although the rise times of the glutamate transients were similar in both genotypes (WT, 15.56 ± 2.6 and RTT, 17.39 ± 1.7 ms, Fig 2Bb, *P* = 0.4, Mann-Whitney-*U*-test), the transients in WT decayed significantly faster (57.6 ± 8.2 ms) than in RTT (99.6 ± 6.7 ms Fig 2Bc, *P*<0.05, Mann-Whitney-*U*-test); again indicating less efficient glutamate uptake in the RTT slices. The 2-fold difference in basal [Glu] can hence be explained by the twice slower uptake in RTT.

Extracellular stimulation of Schaffer collaterals in the organotypic slices (n = 5) evoked glutamate increases of 6.17 ± 2.3 μM (WT), and 8.89 ± 1.7 μM (RTT) in the CA1 area (Fig 2Ca and b, *P*<0.05, Mann-Whitney-*U*-test) with characteristics similar to that of spontaneous glutamate transients. The rise times of these evoked transients were similar in WT (9.56 ± 2.93 ms) and RTT (11.78 ± 1.13 ms) slices (Fig 2Cc, *P* = 0.5, Mann-Whitney-*U*-test). But once again, the glutamate transients decayed faster in WT (44.4 ± 2.0 ms) than in RTT (68.1 ± 3.7 ms, *P*<0.05, Mann-Whitney-*U*-test, Fig 2Cd), which also pointing to a less efficient removal of glutamate from the extracellular space of RTT tissue.

RTT CA1 neurons in addition showed enhanced excitability (37 ± 6.17 Hz) in comparison to WT CA1 neurons (20 ± 2.68 Hz). Stimulations with current steps resulted in the production of significantly more APs in RTT CA1 neurons in relation to WT. Fig 2D presents representative traces generated in response to current injection into CA1 neurons. The cumulative input-output relation in Fig 2Db is obtained from the analysis of recordings of 12 neurons from each genotype (*P*<0.05, Mann-Whitney-*U*-test).

### Repetitive glutamate transients require intact neuronal and synaptic activities

**Fig 3:**
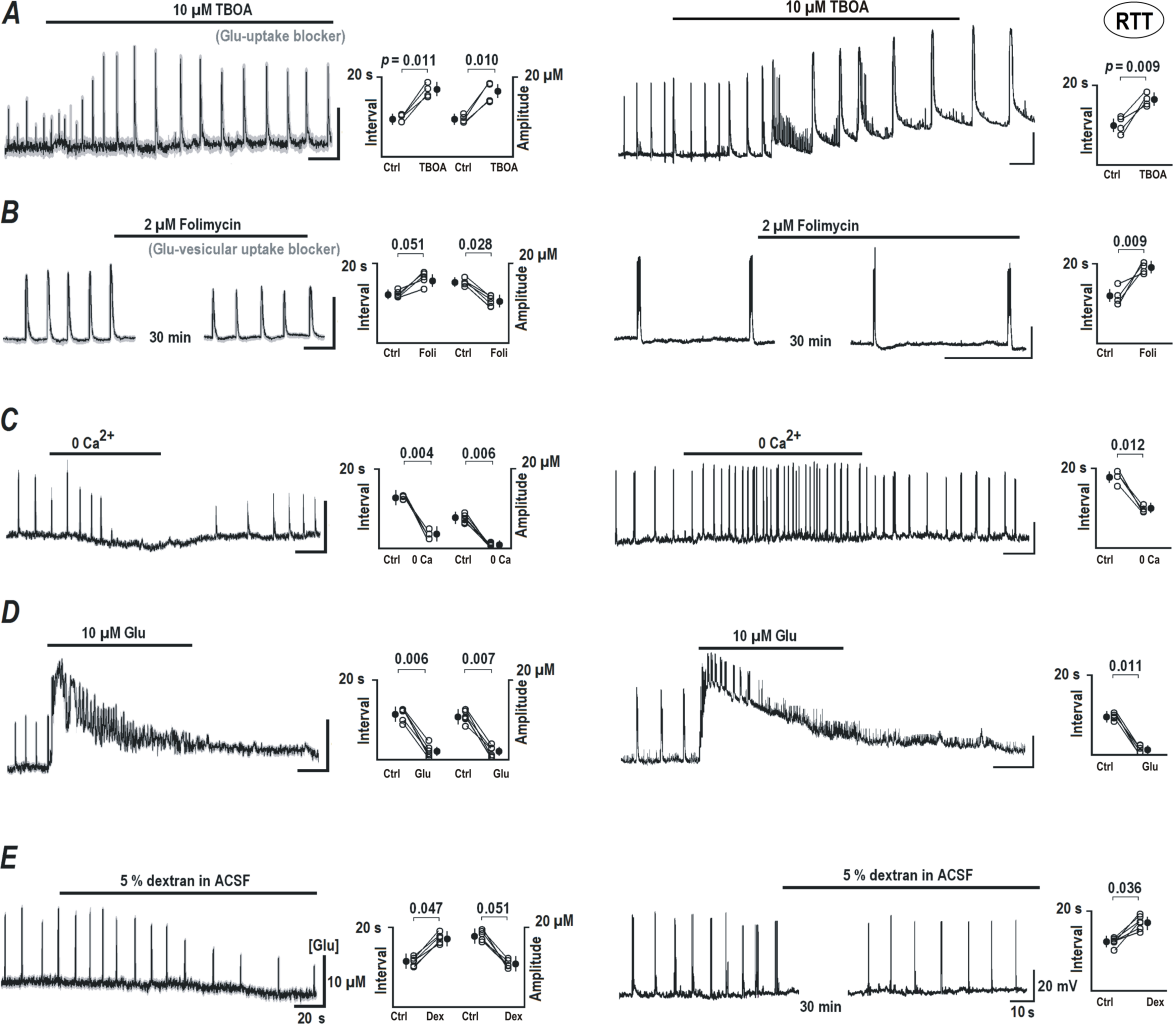
Spontaneous glutamate transients and neuronal activity after blockade of specific pathways of glutamate transport and release. Left panels present glutamate changes, and the corresponding voltage trajectories in whole-cell patch-clamp recordings are shown on the right. ***A***–TBOA (DL-threo-beta-benzyloxyaspartate, 10 μM, a general blocker of glutamate transporters) potentiated spontaneous glutamate spikes and enhanced bursting activity. ***B***—Treatment of the slices for 30 min with 2 μM folimycin (an inhibitor of V-ATPase that prevents refilling of glutamate vesicles) reduced the amplitude of glutamate spikes and decreased their frequency (*left*), in parallel with changes in neuronal activity (*right*). ***C****—*Calcium removal from the bath reversibly inhibited glutamate spikes (*left*), but the neuronal activity increased due to surface potential change (*right*, see also Results section). ***D****–*Brief application of glutamate caused massive long-lasting glutamate increase and neuronal depolarization that eventually subsided. A depression lasted 2–3 min after which the activities fully restored. ***E****–*Application of ACSF containing 5% dextran decreased the amplitude and frequency of glutamate spikes. The statistical significance of differences was evaluated using a Student’s *t* test and corresponding *P* values are listed in group summary.

The data in the previous section indicates a crucial role of glutamate uptake and release to be involved in the generation of glutamate transients in RTT slices. Most experiments are done in this preparation as it showed spontaneous activity much more frequently than WT slices. First, a blockade of the glutamate clearance with TBOA (DL-threo-beta-benzyloxyaspartate, a general blocker of glutamate uptake [1]) reinforced the appearance of glutamate transients (Fig 3A, left trace) as well as the bursting activity (Fig 3A, right trace) in RTT slices. The amplitude of glutamate transients significantly increased from 9.87 ± 1.3 to 17.43 ± 3.02 μM, (Fig 3A, middle graph n = 8, *P*<0.05, Student’s t test). The interval between glutamate spikes increased from 10.13 ± 2.1 to 18.95 ± 3.2 s (Fig 3A, middle graph, n = 8, *P*<0.05, Student’s t test) after TBOA treatment. Presence of TBOA also altered the kinetics of AP firing in the corresponding CA1 neurons in these slices, and the inter-burst interval of APs increased from 12.13 ± 3.1 to 18.75 ± 3.4 s (Fig 3A, right graph, n = 8, *P*<0.05, Student’s t test). Alternatively, Dihydrokainate, a specific blocker of GLT-1 (EAAT-2), elicited similar effects (n = 4, data not shown), in RTT slices. Further experiments, to examine additional pathways proposed to mediate the release and uptake of glutamate in neurons and glia, are summarized in Supporting information in S2 Fig and S2 File.

After the treatment of RTT slices with 2 μM folimycin for 30 min (a specific inhibitor of V-ATPase in the synaptic vesicles), the amplitude of glutamate transients decreased from 17.33 ± 1.9 to 10.04 ± 2.1μM (Fig 3B, left trace and middle graph, n = 6, *P*<0.05, Student’s t test) and the interval between glutamate spikes increased from 13.41 ± 1.3 to 16.07 ± 2.2 s (Fig 3B, middle graph, n = 6, *P*<0.05, Student’s t test). The interval between the bursts increased from 12.65 ± 2.6 to 19.72 ± 2.7 s (Fig 3B, right trace and graph, n = 6, *P*<0.05, Student’s t test). Another inhibitor of vesicular glutamate uptake, bafilomycin (10 μM for 30 min) had similar effects (n = 3, data not shown) on the propagation of glutamate transients.

In nominal calcium-free solution, used to manipulate synaptic release mechanisms, spontaneous glutamate peaks in RTT slices readily decreased from 8.74 ± 2.2 to 1.17 ± 0.8 μM (n = 5, *P*<0.05, Student’s t test), indicating the suppression of calcium-dependent glutamate release machinery (Fig 3C, n = 5). A removal of calcium from the bath enhanced the CA1 neuronal activity and the inter-burst interval reduced from 18.84 ± 2.1 to 10.26 ± 2.0 s (n = 5, *P*<0.05, Student’s t test). This effect on AP frequency can be explained by the hyperpolarizing shift of the activation curves of sodium and calcium conductances in the calcium-free solutions through changes in the neuronal membrane surface potential [34].

Perfusion with 10 μM glutamate produced a large long-lasting glutamate release (22.64 ± 3.2 μM, n = 5, Fig 3D) in the RTT slices. During this increase, the amplitude of the spontaneous transients reduced from 12.17 ± 2.7 to 2.05 ± 1.0 μM (n = 5, *P*<0.05, Student’s t test) but they appeared more frequently (see modeling in Fig 5E below). At the end of extracellular glutamate application the mean inter-event interval of glutamate spikes reduced from 12.43 ± 2.2 to 2.23 ± 0.1 s (Fig 3D left trace and graph, n = 5, *P*<0.05, Student’s t test) and the inter-burst interval of APs in CA1 neurons decreased from 9.39 ± 1.9 to 0.84 ± 0.04 s (Fig 3D, right trace and graph, n = 5, *P*<0.05, Student’s t test).

Ambient glutamate level changes might help to synchronize the activity of network through diffusion in extracellular space. We included dextran (a complex polysaccharide) in the ACSF to retard glutamate spread-out [35] in the slices. The measured viscosity of ASCF with 5% dextran was 1.9 ± 0.2 (n = 12) fold higher than the normal ACSF. This should decrease the glutamate diffusion coefficient by approximately twofold [35]. In dextran-containing ACSF the amplitude of spikes slowly reduced from 17.89 ± 3.0 to 11.91 ± 3.2 μM (*P*<0.05, Student’s t test) and the interval between them increased from 12.77 ± 3.1 to 18.87 ± 3.2 s (*P*<0.05, Student’s t test). Inter-burst intervals increased from 13.14 ± 1.7 to 16.24 ± 1.9 s, *P*<0.05, Student’s t test) (Fig 3E, n = 12).

### Regenerative glutamate spikes require synaptic activity and voltage-sensitive calcium channels

**Fig 4:**
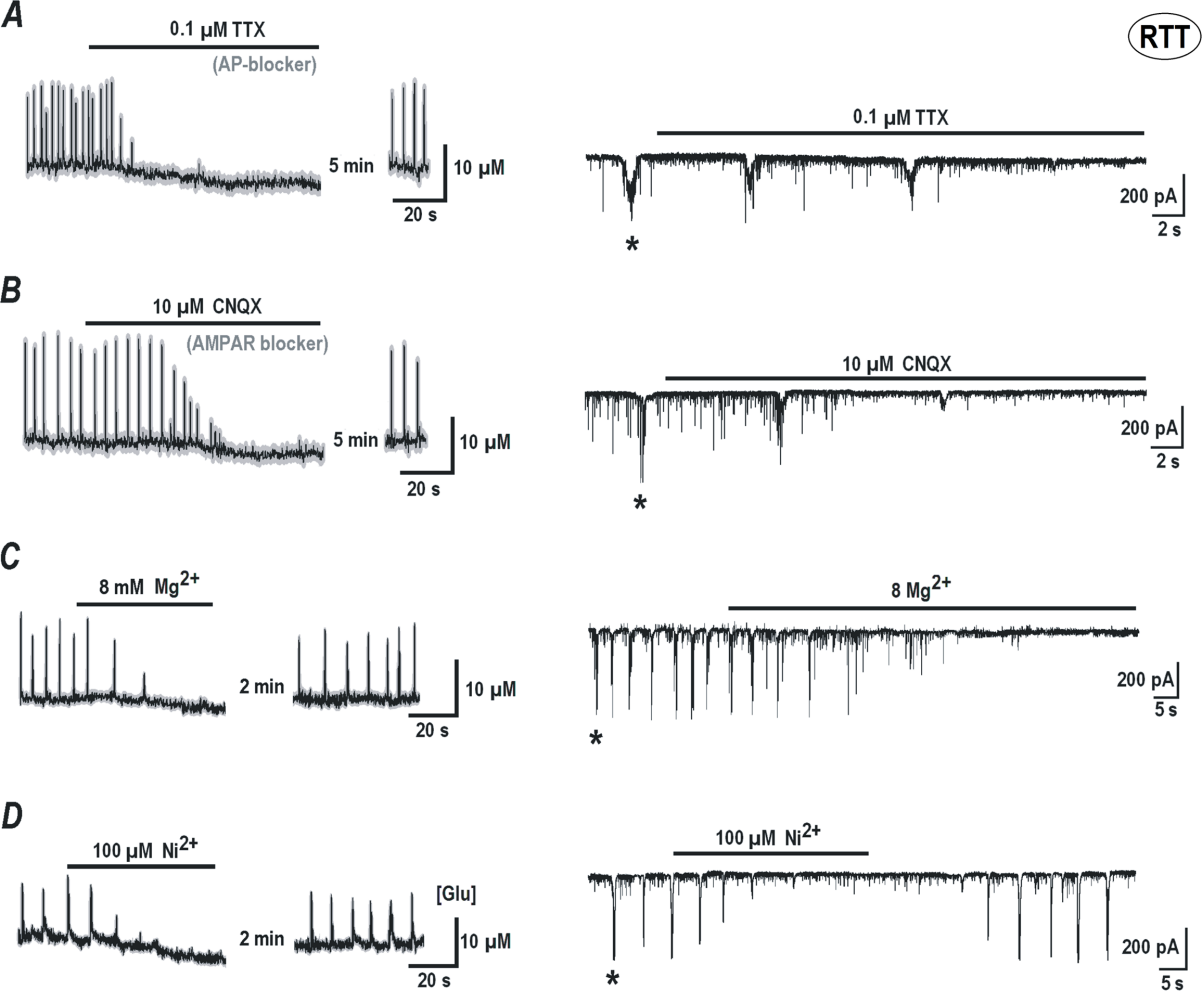
Persistent glutamate spikes require intact neuronal and synaptic activities and calcium influx. Glutamate imaging (*left*) and whole-cell patch-clamp recordings (*right*) show typical responses of CA1 neurons in hippocampal slices from Mecp2 null (RTT) mice. Black thin curves on the left show ambient glutamate changes (averages for 12 cells in the image field) and the thick grey background indicates ± SEM. All blocking effects were reversible and the activities fully recovered after several minutes of wash out. Glutamate spikes were inhibited in parallel with the suppression of synaptic drives, a correlate of AP bursts. The latter are indicated by asterisks in the beginning of each patch-clamp recording (see also Fig 1D). ***A***–TTX (tetrodotoxin, 100 nM, a blocker of the voltage gated sodium channels) suppressed rhythmic glutamate transients, synaptic drives and spontaneous synaptic currents. ***B***—CNQX (Cyano-7-nitroquinoxaline-2, 3-dione, 10 μM, a blocker of AMPA receptors) had similar effects. Elevation of extracellular Mg^2+^ from 1 to 8 mM (***C***) and application of 100 μM Ni^2+^ (***D***) inhibited glutamate transients and synaptic activities. Note small decreases in basal glutamate levels during all treatments.

**Fig 5:**
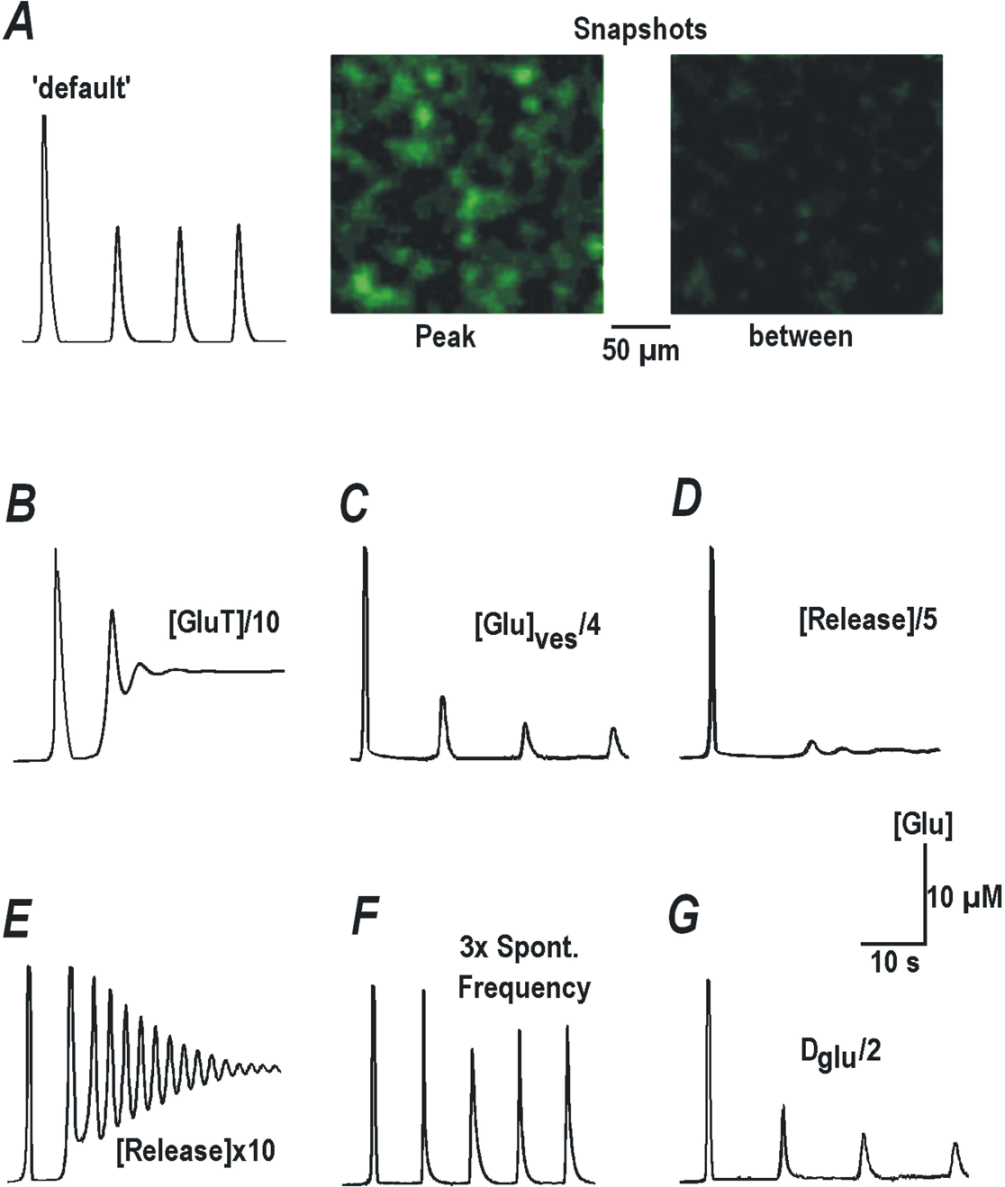
Modeling glutamate-induced glutamate release (GIGR). The traces present mean concentration of ambient glutamate averaged over the 2D-network model. The basic assumptions of GIGR model and the parameters are presented in Methods. ***A***–Fast establishment of rhythmic synchronous glutamate transients within the network. This run was made with ‘default’ model parameter values (see Methods). The two snapshots on the right present spatial distributions of ambient glutamate at the peak of network activity and in between. The following panels show sample simulations made after modification of single model parameter while keeping others at default values. ***B***—Concentration of glutamate transporters (GluT) was decreased 10-fold to mimic a decrease in glutamate uptake rate after TBOA application. This gave rise to an increase in ambient glutamate and interrupted oscillation because all glutamate release sites entered inactivation (refractory) state. ***C***–To simulate the effects of folimycin, which reduces accumulation of glutamate in synaptic vesicles, the glutamate content in vesicles was made 4-fold smaller. This markedly reduced the amplitude and frequency of repetitive glutamate transients (Fig 3B). ***D***–To model calcium-free experiments (Fig 3C), the release rate was decreased 5-fold and glutamate transients rapidly diminished. ***E***–To imitate extracellular glutamate applications. (Fig 4D), the release rate was increased 10-fold. This ambient glutamate slowly elevated and glutamate spikes accelerated but lost their amplitude. ***F***—The frequency of spontaneous release (Eq (3) in Methods) was increased 5-fold. This produced a robust increase in the amplitude and frequency of glutamate transients. ***G***—To mimic the viscosity increase in ACSF with 5% dextran (Fig 3E), the diffusion coefficient of glutamate was made twice smaller. Glutamate transients were decelerated and appeared at lower amplitude.

Generation of spontaneous glutamate transients in RTT slices depended crucially upon neuronal activity; requiring intact sodium and calcium channels as well as intact glutamatergic transmission through AMPA receptors. Tetrodotoxin (TTX, 100 nM), which blocks fast sodium channels and abolishes action potentials, inhibited glutamate transients in RTT slices and the corresponding repetitive synaptic drives, a correlate of AP bursts (Fig 4A, n = 6). The effects were reversible and the previous activities were restored completely after a 5 min-long wash out with fresh ACSF. AMPA receptor blocker CNQX (cyano-7-nitroquinoxaline-2, 3-dione, 10 μM) also reversibly inhibited the glutamate transients and the bursting activity (Fig 4B, n = 12). Spontaneous activity was also suppressed after elevation of extracellular Mg^2+^ from 1 to 8 mM (Fig 4C, n *=* 10), a treatment that is usually used to reversibly suppress synaptic transmission. To note is that, during applications of TTX, CNQX and elevated Mg^2+^, the basal glutamate levels slowly decreased and fully restored after the drugs were washed out. These data allows ascribing synaptically released glutamate as the source driving the regenerative glutamate spikes through glutamate diffusion into the extracellular space (see the data with dextran above, Fig 3E and the model (Fig 5G) below). Additionally, 100 μM Ni^2+^ rapidly and reversibly inhibited glutamate spikes and synaptic drives in the CA1 neurons (Fig 4D, n *=* 8, RTT slices), which indicates the necessity of intact calcium influx in the generation of the glutamate spikes.

### Mechanistic explanation of glutamate spikes

The data in Figs 3 and 4 clearly indicates the putative roles of neuronal activity (experiments with TTX), calcium-dependent (folimycin, 0 Ca^2+^, 0.1 mM Ni^2+^, 8 mM Mg^2+^) glutamatergic transmission (CNQX) and glutamate uptake (TBOA) in the generation and maintenance of glutamate spikes. To analyze each contribution further, we designed a model (Methods) and simulated various experimental conditions in Fig 5.

Brief glutamate transients imply a regenerative process that may be mechanistically similar to the generation of action potentials [36] and calcium-induced calcium release in the heart cells [37] and neurons [38]. Consequently, this phenomenon can be dubbed as ‘glutamate-induced glutamate release’ (GIGR). Although this term has been coined before to explain spreading depression in rat brain slices [11], the role of possible mechanisms have not been specified and examined.

The mechanistic model introduced in Methods does not consider all aforementioned processes explicitly but rather embeds the effects that they can produce. The simulations were made within a two-dimensional network containing synapses placed randomly at a mean distance 1 μm. They are spontaneously activated by synchronous release (induced by AP bursts) and inactivated (desensitized) at higher extracellular ambient glutamate. All runs started with the mean concentration of ambient glutamate, [Glu] = 0.1 ± 0.05 μM, and all glutamate release sites in closed state (scheme (3)). During simulation, all sites then entered the open state almost simultaneously and produced a huge glutamate transient. The sites were inactivated and became refractory. After glutamate levels lowered, a new glutamate peak was generated that occurred nearly simultaneously at all release sites. The pattern of repetitive activity critically depended on the set of chosen model parameters. For default model values, glutamate spikes were generated regularly and synchronously within a whole network at a mean period 10 s (Fig 5A). The distributions of ambient glutamate in the image area at the peak of these responses and between the spikes are shown as insets in Fig 5A. To imitate the experimental data shown in Figs 3 and 4, the model parameters were accordingly changed. In each test only one parameter was modified and the others had the default values. For a decrease in the glutamate uptake rate, to mimic the actions of TBOA, the glutamate concentration stabilized at an elevated level and the oscillations subsided (Fig 5B). When the glutamate content in the vesicles was decreased by 4-fold (presumable result of folimycin action), the amplitude and frequency of glutamate spikes decreased with time (Fig 5C). When the release rate was set 5-fold smaller, to imitate the calcium removal from the bath, the glutamate spikes subsided (Fig 5D). Stimulation of glutamate release increased steady glutamate level that stabilized at a higher value, whereas the oscillations became more frequent (Fig 5E). This run mimicked the changes observed during exogenous glutamate application (Fig 3D). When the rate of spontaneous release was increased (the last term in Eq (1) above), the glutamate spikes attained amplitude and frequency (Fig 5F). A 2-fold decrease in the diffusion coefficient decreased both the amplitude and the frequency of glutamate spikes (Fig 5G) as it was observed in the experiments with dextran (Fig 3E).

The results of the simulations thus provide a simple mechanistic explanation to how a collection of glutamate release sites (synapses) whose activity is regulated in turn by glutamate (through subsequent activation and desensitization of release) can establish a synchronous activity within the network. The release sites communicate through diffusion within extracellular space in the neuropil, which embeds most synaptic connections. The simulations in the model clearly matched experimental observations that showed simultaneous and regular appearance of glutamate spikes in the whole CA1 field of RTT slices.

### T- and R-type calcium channels modulate bursting and synaptic activity and generate glutamate spikes

Glutamate spikes in RTT slices required calcium influx for their generation and robust appearance. Therefore, we next examined the role of different types of voltage-sensitive calcium channels (VSCC) in causing the spontaneous glutamate transients in these slices. Inhibition of N-type channels with ω-conotoxin GVIA and P/Q channels with ω-agatoxin TK completely eliminated glutamate spikes and bursts (both drugs were at 1 μM, n = 3, data not shown). Since these channels are indispensable for glutamatergic transmission [39], these effects, together with that of TTX and CNQX, indicate the strict requirement of glutamatergic synaptic activity in the generation of glutamate spikes. Antagonists of L-type calcium channels (nimodipine and nitrendipine, 50 μM) were less effective and suppressed glutamate spikes and neuronal bursts by around 50% (n *=* 4 for both, data not shown). Ni^2+^ at submillimolar concentrations preferentially blocks T- and R-types calcium channels [40, 41], and in our experiments, 100 μM Ni^2+^ rapidly and reversibly inhibited glutamate spikes and synaptic drives (Fig 4D, n *=* 8) of CA1 neurons.

**Fig 6:**
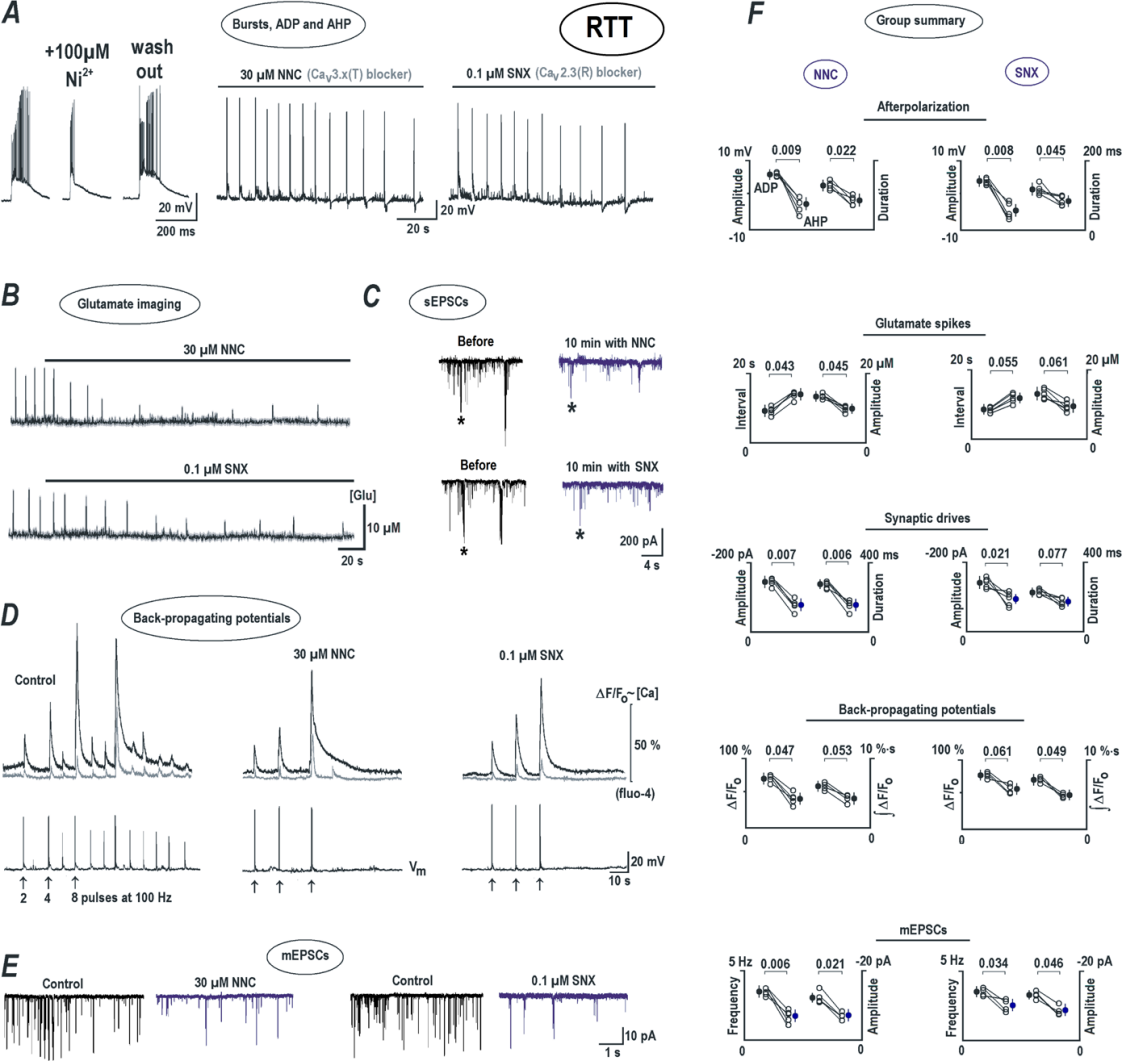
T- and R-type calcium channels modulate glutamate spikes, synaptic and bursting activities. The data presented was obtained in CA1 neurons from RTT animals. In WT cells the effects were similar but weaker due to smaller amplitudes of calcium currents (S3 Fig and S3 File in Supporting information). ***A*–**Current-clamp recordings. The voltage trajectories on the left show the burst before and after addition of Ni^2+^, which blocks both T- and R-type channels, Ca_v_3.2 and Ca_v_2.3, respectively. Note a significant afterdepolarization (ADP) in the control, on the peak of which a series of the actions potentials (a burst) is generated, and its significant (reversible) shortening after Ni^2+^. The two following continuous traces show ADP inhibition by specific blockers of T- and R-type channels, NNC and SNX, respectively. Note that ADP blockade was slowly replaced by afterhyperpolarization, in parallel with decreases in the synaptic and bursting activities. ***B*–**NNC and SNX decrease the amplitude and increase the interval between repetitive glutamate spikes. ***C*–**The blockers diminished the amplitude and frequency of spontaneous excitatory synaptic currents (sEPSC) and suppress the synaptic drives (the former ones are indicated in continuous traces by asterisks). ***D*–**Back-propagating potentials (bAPs) evoked by three brief trains (2 to 8 pulses delivered at 100 Hz as indicated by arrows). Calcium changes were measured with fluo-4 whose relative changes in fluorescence are nearly proportional to intracellular calcium levels. The data is presented for cell soma (black traces) and for apical dendrite 100 μm away from the soma (grey traces). Lower traces show voltage trajectories. In the control, several discharges and calcium increases persisted long after the stimulus. SNX and NNC diminished the amplitude of bAPs-evoked calcium transients and abolished spontaneous voltage and calcium spikes after the stimulations. ***E*–**Miniature EPSCs before and 15 min after addition of calcium channel blockers to the bath (the recordings were made at -70 mV in the presence of 100 nM TTX). ***F*–**Group summary of blocker effects. The data was evaluated before and after application of blockers with Student’s *t* test and corresponding *p* values are listed in graphs. For bAPs-evoked calcium changes, peak amplitudes and the integral of fluorescence changes (a measure of spontaneous afterdischarges) are compared.

T-type (Ca_v_3.x) and R-type (Ca_v_2.3) calcium channels have secondary roles in glutamatergic transmission. They are however important in the generation of bursting activity. A typical burst in the CA1 neurons is established after the first AP during afterdepolarization (ADP) that lasts up to several hundred milliseconds. ADP duration was markedly shortened by 100 μM Ni^2+^ (Fig 6A) treatment. To dissect the role of T- and R-type channels, we used NNC 55–0396, a synthetic drug selective for T-type calcium channels (IC_50_ ≈10 μM). It suppressed the bursting activity, reduced ADP duration from 133.44 ± 8.7 to 87.76 ± 12.1 ms, (*P*<0.05, Student’s t test) and the bursts were then followed by afterhyperpolarization during NNC treatment (AHP, mean amplitude -3.8 ± 0.4 mV (second panel in Fig 6A, n *=* 6).

SNX-482 is a peptidyl toxin that specifically blocks R-type calcium channels (IC_50_ = 30 nM), albeit sometimes incompletely [42]. The effect of SNX on bursting was similar to NNC (initial ADP had amplitude of 4.83 ± 0.6 mV and then converted into AHP with amplitude -4.3 ± 0.5mV, the third panel in Fig 6A). The glutamate spikes in the presence of NNC decreased from 13.82 ± 1.5 to 10.56 ± 1.6 μM (*P*<0.05, Student’s t test) and the interval between the spikes increased from 10.01 ± 2.3 to 14.81 ± 2.4 s (*P*<0.05, Student’s t test, Fig 6B, n *=* 6). NNC suppressed spontaneous synaptic currents. In particular, the amplitude of synaptic drives reduced from 151.34 ± 4.2 to 78.37 ± 6.1 pA and their duration reduced from 272.12 ± 9.7 to 182.45 ± 12.3 ms (both *P*<0.05, Student’s t test). SNX also decreased the amplitude of synaptic drives from 155.08 ± 10.05 to 102.66 ± 8.9 pA and their duration shortened from 226.27 ± 11.05 to 192.03 ± 12.48 ms (*P*<0.05, Student’s t test; Fig 6C, n *=* 6). Neither blocker eliminated the activity completely, which suggests similar and independent contributions of T- and R-type channels to the bursting activity in these cells.

T- and R-type channels are important in the dendritic integration of synaptic signals and can be studied from the kinetics of back-propagating action potentials (bAPs) [43]. In the recordings we combined patch-clamp with intracellular calcium measurements with fluo-4. Brief trains of 2, 4 and 8 pulses of depolarizing current injection (0.7 to 1 nA) at 100 Hz reliably elicited bAPs in CA1 neurons. They were measured electrically at the soma and as calcium changes at the soma and the apical dendrites. Brief trains in the control condition (ACSF without drugs) often induced ‘afterdischarges’ mirrored by calcium changes in the soma and apical dendrites of CA1 neurons. To quantify the effects, we compared the changes in ΔF/F_o_ during and after bAPs. The relative increases in fluo-4 fluorescence are approximately proportional to the intracellular calcium level (see Methods). The group summary on the right in Fig 6D shows calcium changes evoked by bAPs and the integral of the response after the last bAP train, which represents the measure of post-activity. A blockade of T- and R-type calcium channels with NNC (n *=* 5) and SNX (n *=* 5) decreased both variables. The amplitudes of calcium transients during bAPs reduced from 76.14 ± 4.4 to 42.18 ± 4.2% in the presence of NNC and from 82.14 ± 3.6 to 51.32 ± 3.9% (both *P*<0.05, Student’s t test) in the presence of SNX. The integral of the calcium response (area under the curve after bAPs) decreased from 7.01 ± 1.1 to 4.45 ± 1.4%^.^s (NNC, *P*<0.05, Student’s t test) and from 7.6 ± 1.4 to 5.01 ± 1.5%^.^s (SNX, *P*<0.05, Student’s t test) (Fig 6D).

These postsynaptic recordings indicate distinct roles of T- and R-type calcium channels in dendritic processing. These channels may also have an important role at the presynapse [44]. To reveal their contribution at presynapse, we evaluated miniature EPSCs (mEPSCs) before and after application of T- and R-type calcium channel blockers. The mEPSCs of CA1 neurons had mean amplitudes of around 20 pA and frequencies around 5 Hz. The mean amplitude of mEPSCs in RTT decreased after NNC from 15.41 ± 2.7 to 8.85 ± 3.0 pA (n *=* 5) and after SNX from 16.97 ± 1.7 to 10.23 ± 1.8 pA (n *=* 5). The changes are in accordance with the postsynaptic actions. However, the blockers also changed mEPSCs frequency (3.32 ± 0.6 to 2.38 ± 0.8 Hz after NNC and 2.91 ± 0.5 to 2.37 ± 0.7 Hz after SNX) which indicates presynaptic actions of the blockers (Fig 6E, both *P*<0.05, Student’s t test). The rightmost panels in Fig 6F show the statistics of the experiments and compare the effects of T- and R-type calcium channel blockers in RTT slices. Analogous experiments in WT cells are presented as Supporting information in S3 Fig and S3 File.

### Differences in the properties of HCN- and calcium channels in WT and RTT

The low-threshold voltage-sensitive calcium channel (VSCC) and HCN currents might play a major role in determining both neuronal activity and glutamate spikes in CA1 neurons, because they are activated at subthreshold potentials. We isolated either channel currents in the voltage-clamp recordings using intracellular solution containing Cs^+^ + TEA (see Methods). In these experiments, VSCC and HCN currents were elicited by depolarizing and hyperpolarizing voltage steps, respectively.

**Fig 7:**
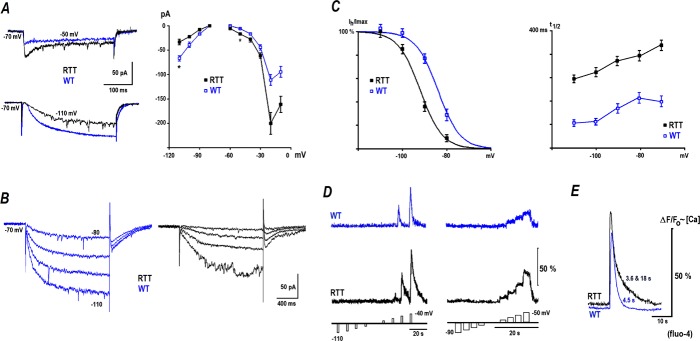
Membrane currents and evoked calcium transients. ***A*–**Sample HCN and calcium currents evoked by voltage steps to -50 and -110 mV, respectively, from the holding potential -70 mV, close to the measured resting potential. CA1 neurons were intracellularly dialyzed with intracellular solution containing Cs^+^ + TEA (see Methods). Under these conditions depolarization-evoked calcium currents (upper panel, the voltage steps to >-60 mV) and hyperpolarization-activated HCN currents (lower panel, the voltage steps to<-80 mV) are clearly isolated. The right graph presents I-V curves for steady state currents in CA1 neurons from WT and RTT animals as indicated. ***B–***HCN currents in WT (blue) and RTT (violet). Note smaller amplitude and slower time-course in RTT cells. ***C***–Potential dependence of activation. The curves were obtained from the tail currents and normalized to maximum (*left*). The half-rise times (*t*_*1/2*_) are shown in the right panel. The differences can be attributed to decreased cAMP levels in RTT [25, 28]. ***D***–Calcium transients during membrane depolarization. The traces indicate calcium changes measured with fluo-4 (100 μM in the pipette) evoked by the voltage step protocols schematically shown under the traces. ***E***–Differences in the voltage-evoked calcium transients. Depolarization to 0 mV for 1 s evoked calcium increases in WT CA1 neurons (blue) that were smaller and faster than those in RTT cells (violet). Subsequent decay to the resting level was described by a single exponential in WT and in RTT an additional slower exponential appeared (the values are listed near the respective curves).

Fig 7A compares sample currents evoked by voltage steps to -50 mV (calcium) and to -110 mV (HCN) from the holding potential of -70 mV. The mean amplitudes of the calcium currents at -50 mV were -12.67 ± 1.4 pA (WT) vs. -24.37 ± 1.7 pA (RTT), and HCN currents at -100 mV were -73.67 ± 4.6 pA, WT; vs. -29.08 ± 4.7 pA, RTT (n = 10, *P*<0.05, Student’s t test for both). The same differences were observed in the acute slices (S1 Fig and S1 File in Supporting information).

The main differences in I-V the curves of WT and RTT CA1 neurons concerned the subthreshold ranges of voltages. This could be critical to determine the intrinsic bursting activity of CA1 neurons. Substantial activation of calcium currents at potentials <-50 mV (Fig 7A) is itself sufficient to explain hyperexcitability in RTT (Fig 2D). Presumed excitability should be further enhanced due to a shift of activation of HCN currents to hyperpolarizing direction and strengthened by slower activation of HCN channels in RTT (Fig 7C). The changes in properties of HCN channels in RTT can be explained by a decreased cAMP level, reported previously in the acute hippocampal slices [28] and directly measured in the brainstem with a neuronal Epac-based sensor [25].

Further differences in cellular signaling between CA1 neurons in WT and RTT were revealed by calcium imaging experiments. In RTT CA1 neurons, brief voltage steps (500 ms) evoked fast and large calcium transients. A voltage step to -30 mV induced changes in fura-2 fluorescence of ΔF/F_0_ = 74.90 ± 2.4 (RTT) vs. 55.96 ± 2.7% (WT) (the left panel in Fig 7D, n = 8, *P*<0.05, Student’s t test). Longer lasting subthreshold voltage steps to -50 mV gave ΔF/F_0_ = 28.16 ± 2.7 (RTT) vs. 16.10 ± 2.6% (WT), (the right panel in Fig 7D, n = 8, *P*<0.05, Student’s t test). These results also shows bigger calcium fluxes in RTT neurons at subthreshold voltages, in line with the whole-cell recordings (Fig 7A).

Non-selective HCN channels are shown to mediate calcium influx in HEK293 cells and dorsal root ganglion (DRG) neurons, which was suggested as a possible mechanism to reinforce secretion [45]. These described effects only became significant for potentials <-120 mV and developed on a scale of tens of seconds, conditions that are not physiological. Of note, in our hands (Fig 7D), CA1 neurons did not reveal measurable calcium increases upon hyperpolarization to -110 mV for 10 s.

The brainstem RTT neurons had elevated calcium levels that stem from impaired calcium homeostasis [24]. In CA1 cells, the mean resting calcium measured with fura-2 in eight slices was bigger in RTT (97 ± 17 nM) vs. WT (67 ± 12 nM, *P*<0.05, Mann-Whitney-*U*-test). The mean amplitudes of peak calcium evoked by 1 s-long depolarization to 0 mV were 297 ± 25 nM in WT vs. 367 ± 32 nM in RTT (n *=* 12 in three different preparations from respective animal types, *P*<0.05, Mann-Whitney-*U*-test). Calcium decay to the resting level in WT was described by a single exponential with a mean time-constant of 4.2 ± 0.3 s, whereas for RTT it had a double-exponential waveform with decay time constants of 3.9 ± 0.3 and 17.2 ± 1.2 s for the fast and slow components respectively (Fig 7E).

### HCN channels modulate bursting and synaptic activities and shape glutamate spikes

**Fig 8:**
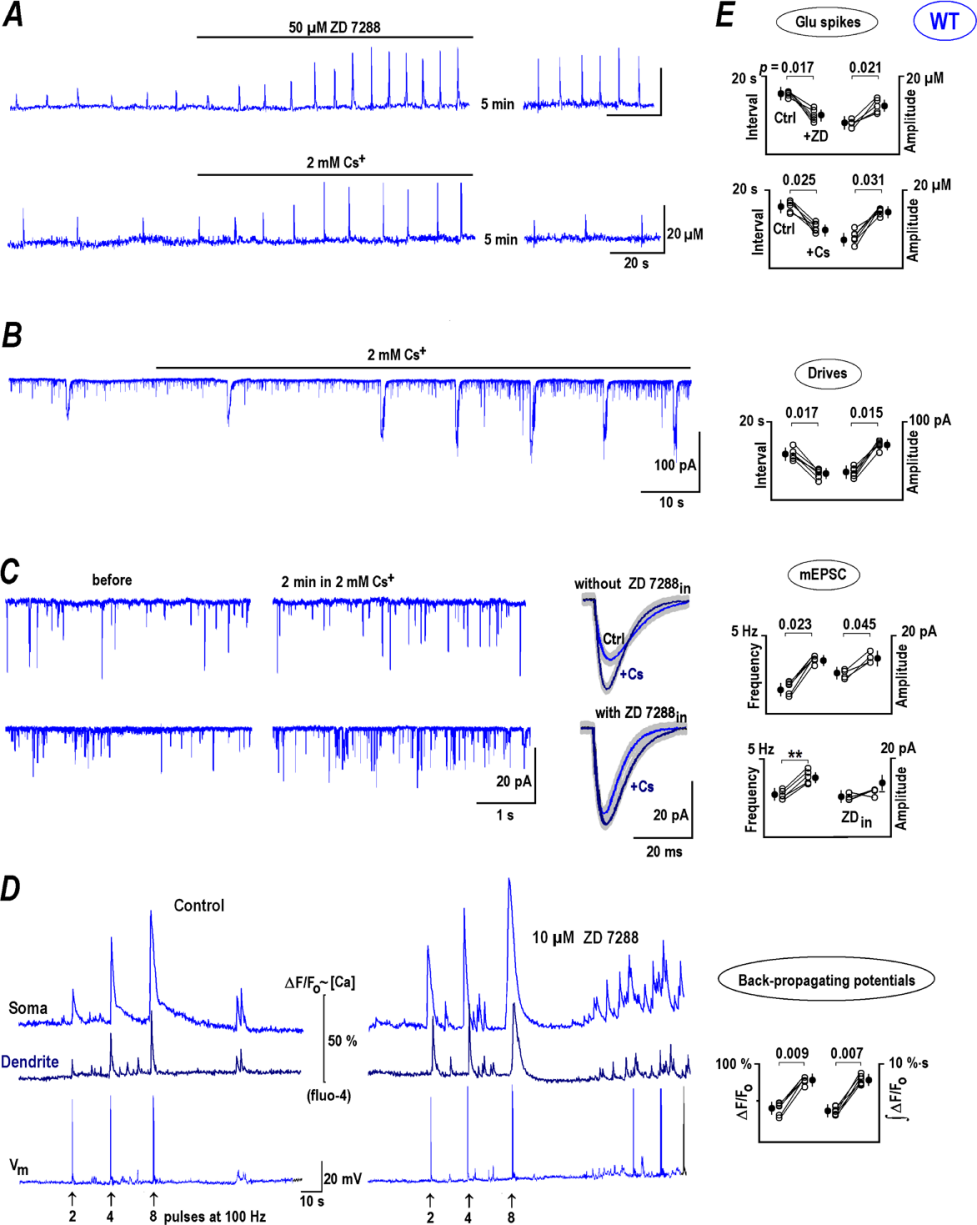
HCN channel activity shapes glutamate spikes and synaptic currents. Presented data was obtained in WT CA1 neurons, where HCN conductance was bigger than in RTT and the effects of blockers were more expressed (for typical experiments in RTT and mean data, see S4 Fig and S4 File in Supporting information). ***A*–**Glutamate spikes had bigger amplitudes and appeared more frequently after application of HCN blockers. ZD 7288 acted irreversibly and enhanced activity, whereas the potentiating effect of Cs^+^ reversed after 5 min of wash out. ***B*–**Potentiation of synaptic drives by Cs^+^. Shown are the changes in the holding current at -70 mV after addition of 2 mM Cs^+^ to the bath. ***C*–**Miniature EPSCs before and after addition of 2 mM Cs^+^ to the bath. The pairs of traces were obtained from the two representative cells dialyzed with K^+^ containing intracellular solution with and without 50 μM ZD 7288, respectively. The recordings were made at -70 mV in the presence of 100 nM TTX. The increase in the frequency and amplitude by extracellular Cs^+^ in the control (without ZD_in_) indicates the effects of pre- and postsynaptic HCN blockade, respectively. To distinguish between them, the neuron was dialyzed with 50 μM ZD 7288 for 15 min. Extracellular Cs^+^ then significantly changed mEPSC frequency but not the amplitude, which demonstrates pure presynaptic effects in this case. The traces in insets are the means of 20 to 30 single mEPSCs before and 2 min after Cs^+^ wash in and grey background indicates ±SEM. In the control Cs^+^ prolonged the decay of mean mEPSC. With ZD 7288 inside the decay time-constant was the same and the amplitude slightly increased, in accordance with pure presynaptic actions only. ***D*–**Back-propagating potentials. The pulse protocol was the same as described in Fig 6D. Note the calcium changes in the soma and apical dendrites mimicked the membrane discharges after the stimuli. Extracellular ZD 7288 potentiated both activities. ***E***—Group summary. The data was evaluated before and after drug applications with a Student’s t test with corresponding *P* values and are listed at the top of the panels.

The role of HCN channels in determining neuronal activity can be dichotomous. The HCN current provides a depolarization drive at voltages more negative to its reversal potential (-40 mV, [46]). It also decreases membrane resistance, and this shunting effect dampens membrane excitability. To distinguish between the effects, we first analyzed WT CA1 neurons, because they had bigger HCN conductance (Fig 7). Fig 8 summarizes the data obtained in WT and S4 Fig and S4 File in Supporting information complements them for RTT. After a blockade of HCN channels, glutamate transients, that were usually small and weakly expressed in WT, gained amplitude and appeared more frequently (Fig 8A). The irreversible blocker of HCN channels, ZD 7288, reinforced the rhythmic activity in CA1 neurons. As a result, the amplitude of glutamate spikes increased from 7.82 ± 2.2 to 14.72 ± 2.3 μM (n = 5, *P*<0.05, Student’s t test) and the intervals decreased from 16.37 ± 1.9 to 12.1 ± 1.7 s (*P*<0.05, Student’s t test). The effect of the irreversible blocker ZD 7288 persisted long after wash out. Additionally, HCN blockade with extracellular Cs^+^ (2 mM) also facilitated glutamate spikes. Their amplitude increased from 7.39 ± 2.0 to 17.30 ± 2.1 μM, n = 5, *P*<0.05, Student’s t test) and the intervals decreased from 16.07 ± 1.9 to 10.27 ± 1.8 s (*P*<0.05, Student’s t test). The Cs^+^ effect was rapidly reversed within 2 min of wash out. Both Cs^+^ and ZD 7288 (Fig 8B, n = 4) potentiated sEPSC and reinforced synaptic drives. Their amplitude elevated from 40.13 ± 4.2 to 69.63 ± 4.1 pA and the interval decreased from 12.24 ± 1.6 to 7.23 ± 1.4 s (*P*<0.05, Student’s t test).

To gain insight into the pre- and postsynaptic role of HCN channels, we examined the effects of blockers on miniature synaptic currents. Two minutes after the addition of 2 mM Cs^+^ to the bath (n = 4), the amplitude of mEPSCs increased markedly from 10.68 ± ± 1.8 pA to 16.74 ± 2.6 pA (*P*<0.05, Student’s t test) and their frequency changed from 1.81 ± 0.5 to 2.82 ± 0.4 Hz (Fig 8C). HCN channels are expressed pre-and postsynaptically in the neurons. To discern between the pre- and postsynaptic effects of HCN blockade, we applied 2 mM Cs^+^ to four CA1 cells after 15 min intracellular dialysis with 50 μM ZD 7288, to block postsynaptic HCN channels. The average mEPSC (inset in Fig 8C) had a slightly increased amplitude after Cs^+^ from 10.34 ± 1.4 to 11.64 ± 1.7 pA (*P* = 0.54, Student’s t test). The mEPSC waveform was not modified but the frequency increased from 2.58 ± 0.4 to 3.17 ± 0.3 Hz (*P*<0.05, Student’s t test, respective group summaries are vertically arranged on the right in Fig 8E). Thus, a blockade of HCN channels in postsynaptic cells with intracellular ZD 7288 unmasked their effects at presynapse. Such effects resemble those observed in the entorhinal cortex, where HCN channels dampen presynaptic activity through inhibition of T-type channels, which leads to the tonic suppression of glutamate release [47].

Postsynaptic modulations of HCN channels in CA1 are also important in processing synaptic inputs through dendritic integration [48, 49]. To test the role of postsynaptic HCN channels, we examined bAPs and the corresponding changes in calcium signals before and after ZD 7288 treatment in six cells (Fig 8D). 15 min after dialysis with ZD 7288, bAP-induced calcium increases changed ΔF/F_0_ from 48.33 ± 3.7 to 84.73 ± 3.6% (*P*<0.05, Student’s t test). A long-lasting potentiation of neuronal activity evoked by brief trains of bAPs was also markedly reinforced and the integral ΔF/F_0_ after the last bAP increased from 3.93 ± 0.4 to 8.84 ± 0.3%^.^s (*P*<0.05, Student’s t test). The results of HCN inhibition in CA1 neurons from RTT animals are presented in S4 Fig and S4 File in Supporting information. The results of the experiments closely match a concept that HCN channels contribute to the postsynaptic dendritic integration and presynaptic glutamate release.

## Discussion

Rett syndrome is a neurodevelopmental disease in children characterized by epileptic episodes. Enhanced neuronal excitation is thought to represent one of the intrinsic factors leading to or manifesting this abnormality. The data presented in this manuscript shed new light on the origin of enhanced excitability in the hippocampus of the RTT syndrome mouse model. The results demonstrate altered glutamate homeostasis in the hippocampus of Mecp2^-/y^ mice. The main observations of this study are as follows: (a) glutamate levels in RTT hippocampal slices are bigger and decay longer indicating slower glutamate uptake than in WT; (b) we frequently observed regenerative glutamate transients (peak, 10 μM; frequency 0.1 Hz) in RTT, whereas such activity was virtually absent in WT; (c) the glutamate transients appeared synchronously in the CA1 area and matched the bursting activity of CA1 neurons, (d) the glutamate spikes required calcium influx, intact glutamatergic transmission and depended upon glutamate diffusion within extracellular space; (e) we designed a novel model to describe glutamate-induced glutamate release and the simulations neatly reproduced the respective experimental data; (f) T- and R-type calcium and HCN channels showed different expressions and voltage-dependent properties in RTT and modulated spontaneous glutamate transients.

Glutamate imaging, in its present status, has both advantages and disadvantages. It delivers important data on ambient glutamate levels within a slice and their changes at millisecond time-scale, which was previously impossible. The iGluSnFR sensor is targeted to the plasma membrane and exposed extracellularly [12, 50]. The network morphology of astrocytes and neurons with different morphological appearances are well depicted by correspondingly targeted sensors (Fig 1A). This, however, does not allow the precise localization of the source of extracellular glutamate, because the plasma membranes of neurons and astrocytes are separated by less than 0.1 μm [6], beyond the diffraction limit of custom imaging. Better spatial resolution could be potentially revealed by confocal, two-photon or STED microscopies. But the methods mostly improve radial resolution. The maximum axial resolution in brain slices is expected to be >1 μm and glutamate signals from the densely packed presynaptic sites (mean distance 1 μm) should be immensely smeared. Spatial improvement in these methods is also achieved at the cost of the temporal resolution. This posits the glutamate imaging during exocytosis of single vesicles as a challenging task with formidable technical difficulties. Perhaps it can be realized only in isolated neurons or astrocytes in culture, where out-of-focus effects are minimal and cell borders are well defined. Despite the aforementioned caveats, AAV-targeted glutamate sensors present a real improvement over previous approaches to assess glutamate levels within intact brain tissue. Methods such as selective microelectrodes, brain dialysis, NMR etc. operate at much bigger space and slower time scale and the information retrieved is severely limited.

It is commonly accepted that each exocytosis step at glutamatergic synapse releases approximately 1 mM glutamate within 1 ms [30]. This is thought to drive the sensor signals to saturation but our calibrations (Fig 1B) show that reliable ambient glutamate levels can be unambiguously determined from the dose-response curve equation (see Methods and Fig 1A). We do yet believe that glutamate imaging provides only the lower estimates due to unavoidable spatial and temporal filtering during image acquisition.

To the best of our knowledge, the occurrence of repetitive glutamate transients in brain tissue has not been reported before. In CA1, as well as in CA3, areas of the organotypic slices from RTT animals, we frequently observed repetitive glutamate transients (Fig 1E). Ambient glutamate was elevated in RTT, which we explain by less efficient glutamate uptake (Fig 2). Higher extracellular glutamate and its frequent spontaneous increases can induce tonic activation of specific signaling pathways (see below). Their action(s) may converge and result in increase in subthreshold calcium conductance and decrease in the activity of HCN channels in RTT. For example, glutamate receptors produce long-term potentiation of calcium currents in isolated sensory and hippocampal neurons [51]. VSCC and HCN channels show opposite changes in RTT CA1 neurons (Fig 7). Both effects can predispose the neurons to hyperexcitability, which facilitates spontaneous glutamate release and may make the RTT hippocampus prone to epileptic seizures, one of the hallmarks of Rett Syndrome [14–17]. The effects are intrinsic to RTT as in the acute slices similar differences between RTT and WT cells are observed (S1 Fig and S1 File in Supporting information).

Previous studies suggested aberrant glutamate handling in RTT [18–20], supporting the concept that chronically elevated glutamate levels influence disease progression. Excessive glutamate production is attributed to Mecp2-null microglia [19] and abnormal clearance of glutamate by astrocytes [18]. The two studies have been performed in cultured cells, and the effects need to be confirmed in the intact brain tissue. EEG and *in vivo* measurements of brain glutamate in RTT model mice also indicate abnormal activity-dependent glutamate changes in the frontal cortices and relate them to severe sleep dysfunction [20].

Our data points to presynapse as the origin of elevated glutamate in RTT. Analysis of other possible source such as astrocytes being able to release glutamate gave negative results. After blockade of hemichannels and gap junctions, anion channels, purinergic P_2X_ receptors, and glutamate transporters operating in reverse mode, cysteine-glutamate exchange and Ca^2+^-induced vesicular release [2, 3], the glutamate transients were only modulated but not eliminated (S2 Fig and S2 File in Supporting information). On the other hand, inhibition of neuronal firing and synaptic activities readily abolished the generation of rhythmic glutamate transients (Fig 4). Based on these evidences we speculate that the source of glutamate is neuronal. It likely appears and accumulates in the interstitial space after presynaptic release during intense neuronal activity.

Glutamate transients observed in this study can be related to glutamate waves that underline the spreading depression within the rat brain cortex [11]. We propose that glutamate release in CA1 area is initiated spontaneously and then subsequently inhibited at high glutamate concentrations. A new cycle begins after complete recovery of the release machinery from the refractory state. Mechanistically, this model involves two elements–activatory and inhibitory gates. The proposed model mechanism has two striking parallels in physiology–the generation of action potentials [36] and the induction of calcium-induced calcium release [37, 38]–with the major variables set as membrane voltage and intracellular calcium, respectively. This novel concept of glutamate-induced glutamate release is formalized in Methods. The simulations in the two-dimensional network show that even such a rudimentary scheme is capable of predicting the generation of synchronous repetitive glutamate spikes and their persistence. These simulations efficiently reproduce repetitive glutamate release patterns (Fig 5) observed in the experiments (Figs 3 and 4). This includes the modifications of regenerative glutamate transients after inhibition of glutamate uptake, activation/inhibition of glutamate release; and retardation of glutamate diffusion.

Rhythmic and synchronous activity within CA1 networks seemingly requires persistent support by extracellular glutamate, released from the presynaptic endings that facilitate excitation at neighboring synapses and neurons [30]. The identity of (sub) cellular receptors and signaling pathways that may act as hypothetical activatory and inhibitory gates in the spontaneous glutamate release is yet to be established. One plausible candidate is presynaptic kainate receptors [52–54] that exert either stimulatory or inhibitory effects at low and high glutamate levels, respectively. Another candidate is the Group I metabotropic glutamate receptors, mGluR1/5 [35] which have low affinity to glutamate. mGluR1/5 potentiates R-type calcium channels that can switch AP afterhyperpolarization with an afterdepolarization, which reinforces the bursting activity [40]. Fig 6A presents a reverse transition of the above mentioned mechanism, where the blockade of T- and R- type calcium channels converts afterdepolarization to afterhyperpolarization. The examination of possible functional role(s) of kainate receptors and mGluR1/5 in the generation of glutamate spikes requires further detailed studies.

Hyperexcitability can be well explained by specific alterations in the subthreshold membrane VSCC and HCN conductances in RTT neurons. Preponderance of epilepsy has already been associated with a loss of expression and function of HCN channels [55, 56]. These channels are highly expressed in various brain regions and function as ‘voltage-absorber’ to diminish intrinsic excitability of pyramidal neurons [57]. In CA1 neurons they are also localized at the apical dendrites where the majority of excitatory synaptic input occurs. Their activity at resting and subthreshold potentials acts as an inhibitory drive due to the decrease in dendritic input resistance. In this way HCN channels are able to constrain the CA1 distal dendritic calcium spikes [48] and the efficacy by which AP trains back-propagate into the dendrites and trigger dendritic spikes (Fig 8D).

VSCC and HCN currents show significant differences between CA1 neurons from WT and RTT animals. In RTT, the calcium currents are bigger and HCN currents are smaller in amplitude. The voltage dependence of VSSCs and HCN currents was shifted into the hyperpolarization direction in RTT CA1 neurons. This shift triggers bigger VSSC currents and smaller HCN currents, and has enhancing effect on the excitability of RTT CA1 neurons. Increased calcium current has a direct effect on the excitability of these neurons, whereby, entry of more calcium ions induces neuronal depolarization and excitation. Smaller HCN currents dispose these neurons to further depolarization by being less effective in shunting the membrane resistance at near resting membrane potentials. HCN channel activity is modulated by cAMP and the data is congruent with previous observations of lower cAMP levels in RTT [25, 28].

The role of HCN channels in the regulation of the excitatory-inhibitory imbalance is observed in other autism spectrum disorders such as Fragile X Syndrome. HCN channels appear to have a lower expression in the dendrites of Fmr1^−/y^ neurons and are shown to increase dendritic excitability [58]. Presynaptic HCN channels participate in hippocampal maturation and network responses [59], showing developmental plasticity in axonal and presynaptic compartments and consequently modulate synaptic efficacy. Since the presynaptic expression and function of HCN channels is often extinguished with maturation of the brain, modulation in the expression of these channels may be relevant to RTT, where the many symptoms are shown to be established during early development [60, 61].

Changes in the HCN channel function may not be the sole factor in causing anomalous excitability in RTT. Calcium channels of T (Ca_v_3.2) and R (Ca_v_2.3)—type are functionally present in the dendrites of CA1 pyramidal neurons and contribute to the integration of incoming signals to shape bursting activity [62]. Calcium spikes evoked by back-propagating action potentials can excite the soma recurrently through a ping-pong mechanism [63], and evoke a new AP in the axon initial segment that travels to synapses and excites neighboring neurons. Presynaptic plasticity depends on subthreshold calcium increases (Fig 7D) and can modulate subsequent spike-evoked transmission [43, 64]. Pre- and postsynaptic effects of HCN and VSCC channels often converge [65]. A net result could be the recurrent activity within the hippocampal network that is essential for spontaneous glutamate release.

We propose that, diminished activity of glutamate transporters in RTT leads to substantial increase in ambient glutamate levels (Fig 2Ab), which establishes conditions for GIGR. This is supported by modeling experimental data shown in Fig 5. In particular, diminished glutamate release (Fig 5C and 5D) and impediment of glutamate diffusion dampens glutamate spike propagation, in accordance with the pharmacological data (Fig 3). Blockade of spontaneous activity (not shown in Fig 5) abolishes glutamate spikes, as indicated by the experiments with TTX (Fig 4A) and calcium channel blockers (Fig 6). The effects of sodium channel and AMPA inhibitions (Fig 2A and 2B) clearly show the importance of intact glutamatergic transmission in the maintenance of regular glutamate spike. Whereas, increase in spontaneous release (Fig 5E), and inhibition of HCN channels evidently enhances glutamate spikes generation. Blockade of cysteine-glutamate antiporter in astrocytes with 100 μM sulfasalazine (S2 Fig and S2 File) also resulted in the enhancement of glutamate spikes, but the underlying mechanism was not studied further in this study.

Our former [15, 23–25] and the present studies focus upon postnatal neurons (P10 to P30). The Rett Syndrome was formerly thought to set on during late postnatal development (from P40 on). The recent evidence indicates that RTT symptoms are present at the earliest stages of brain development and produce a phenotype that arises from the pleotropic effects of MeCP2. It functions very early and continues to be expressed into adulthood, leading to the aberrations that include diverse signaling, transcriptional, and epigenetic mechanisms [66]. Net neurophysiological effects in RTT are the imbalance of neuronal excitation and inhibition in neuronal networks and deregulation of activity-dependent mechanisms [67]. Disturbances in the expression of ionic channels and glutamatergic receptors appear around the perinatal age and play an important role in adulthood regulating cerebral cortex development [68]. The delay in maturation within the developing Mecp2 null cortex may stem from deranged mechanisms of cell fate refinement [69, 70].

In summary, present data complements these evidences by showing altered activities of HCN, T- and R-type calcium channels in RTT. They may represent novel etiological candidates to make the Rett brain more prone to epileptogenesis. These channelopathies, in combination with diminished glutamate uptake in RTT, may co-operate to establish repetitive activity in the form of regular glutamate spikes. Such regenerative glutamate release has never been reported in the nervous tissue before, and we aim to achieve more insight into the key causative mechanisms and find their further specific neurological implications.

## Supporting information

S1 Fig Characteristics of CA1 neurons in the acute slices. Recordings were made from slices prepared from 4- to 5-weeks-old WT and RTT animals that approximately corresponded to a postnatal age of organotypic slices examined. The traces from WT and RTT are differently colored (blue vs. black, respectively). ***A*–**First trace in each panel shows sample responses of CA1 cells to the same current injection and spontaneous bursts. The graph on the right presents input-output relationships for acute slices from WT and RTT animals. The data were collected from 8 cells in 3 different preparations.
***B*–**Spontaneous and miniature EPSCs. Shown are sample episodes recorded at -70 mV in ACSF before (upper traces) and 15 min after addition of 100 nM TTX to the bath (lower couple of traces). Mean frequencies and amplitudes were obtained as described in Methods and evaluated with a Mann-Whitney-*U*-test with confidence levels *P* values listed in the graphs. Synaptic drives (indicated by asterisks) in sEPSC recordings were excluded from the analysis. ***C*–**The neurons were dialyzed with intracellular solution contained Cs^+^ + TEA (see Methods). Under these conditions the calcium current evoked by depolarization (upper panel) and HCN current activated during hyperpolarization step (lower panel) were clearly isolated. I-V curves for steady state currents are shown on the right. Mean values were obtained from 12 cells patched in slices from RTT and WT and the bars indicate ±SEM. The currents were not normalized to membrane area, because the capacitances in measured cells were around the same.
(TIF)

S2 Fig Modification of glutamate spikes after blockade of glutamate release and uptake. ***A*–**Thapsigargin (inhibitor of SERCA that mediates calcium uptake into internal stores) transiently potentiated glutamate spikes in ACSF that was followed by depression. Glutamate spikes recovered to the control values after the drug was washed out. ***B***–Suppression of glutamate spikes in calcium-free solutions was countered by thapsigargin, but the effect was short-lasting. The data in these two experiments indicate dependence of glutamate spikes on intracellular calcium that is transiently released from internal stores after SERCA inhibition by thapsigargin. ***C—***DIDS (4, 4’-Diisothiocyano-2, 2’-stilbenedisulfonic acid, an inhibitor of anion exchange reported to mediate glutamate release from astrocytes) slightly inhibited the amplitude and frequency of glutamate spikes. ***D–***Sulfasalazine, a blocker of Cys/Glu transporter reported to release glutamate from astrocytes, enhanced the amplitude and frequency of glutamate spikes. ***E***, ***F***–Carbenoxolone (a blocker of gap junctions formed by connexins) and probenecid (a blocker of hemi-channels transporting small organic anions) decreased the amplitude and frequency of glutamate spikes. All traces were obtained in CA1 area of RTT animals. The data were evaluated before and after applications of blockers with a Student’s *t* test. The corresponding *P* values are listed in group summary.
(TIF)

S3 Fig T- and R-type calcium channels modulate glutamate spikes, bursting and synaptic activities in WT. The data were obtained in CA1 neurons from organotypic slices derived from WT animals (the results of experiments in RTT slices are presented in Fig 5 in the main text). ***A*–**Current-clamp recordings from CA1 neurons. The traces on the left show inhibition of afterdepolarization (ADP) and bursting activity by Ni^2+^. The next panels on the right present actions of T- and R-type channel blockers, NNC and SNX. They suppressed ADP and unmasked afterhyperpolarization (AHP). The blockade was accompanied by decreases in the synaptic and bursting activities. ***B*–**T- and R-type channel blockers decreased the amplitude and frequency of glutamate spikes. ***C*–**Spontaneous EPSCs before and 10 min after application of T- and R-type blockers (the recordings were made in ACSF at the holding potential -70 mV). Both EPSCs and related synaptic drives (indicated by asterisks) were suppressed. Group summary is presented on the right and evaluated with a Student’s *t* test and corresponding *P* values are listed in the graphs.
(TIF)

S4 Fig The effects of HCN channels in RTT slices. ***A*–**HCN blockers augmented the amplitude and frequency of glutamate spikes. ZD 7288 actions were irreversible and did not recover during wash out, whereas Cs^+^ effects reversed fully after wash out for 5 min. ***B*–**The bursting activity was reinforced by Cs^+^ and increased the duration of bursts and decreased the interval between them. The data were evaluated before and after applications of blockers with a Student’s *t* test and corresponding *P* values are listed in group summary.
(TIF)

S1 File Electrophysiology of CA1 neurons in acute slices. (DOCX)

S2 File Analysis of additional signaling pathways influencing glutamate transient propagation. (DOCX)

S3 File Role of T- and R-type calcium channels in WT CA1 neurons. (DOCX)

S4 File HCN channel blockade enhance the spontaneous glutamate transients in RTT slices. (DOCX)
