# Supplementary figures and images for: Regenerative glutamate release in the hippocampus of Rett syndrome model mice
Source: PLoS One. 2018 Sep 26;13(9):e0202802. doi: 10.1371/journal.pone.0202802 (PMC6157837; doi:10.1371/journal.pone.0202802)

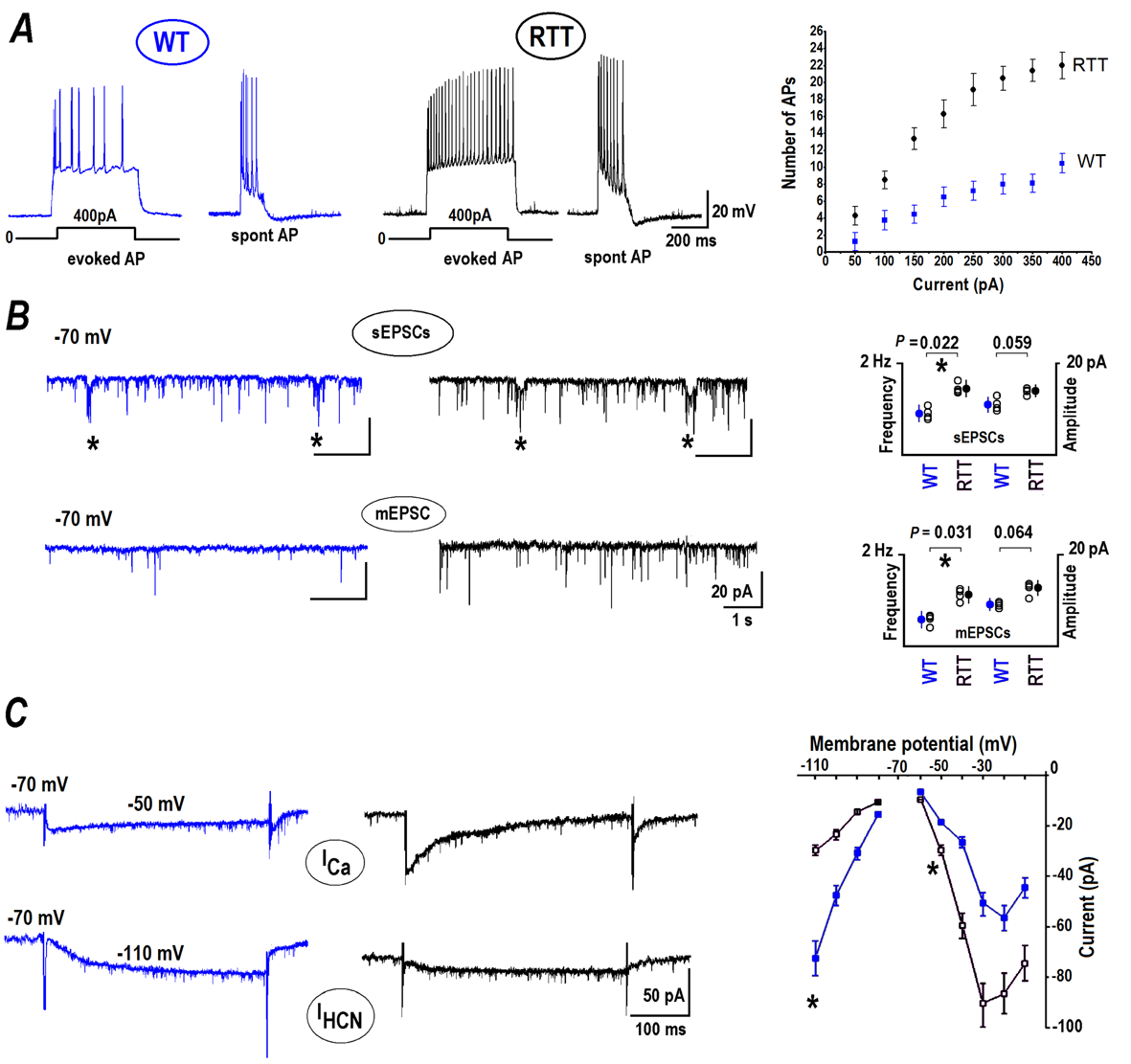

Supplement: S1 Fig — Recordings were made from slices prepared from 4- to 5-weeks-old WT and RTT animals that approximately corresponded to a postnatal age of organotypic slices examined. The traces from WT and RTT are differently colored (blue vs. black, respectively). A–First trace in each panel shows sample responses of CA1 cells to the same current injection and spontaneous bursts. The graph on the right presents input-output relationships for acute slices from WT and RTT animals. The data were collected from 8 cells in 3 different preparations. B–Spontaneous and miniature EPSCs. Shown are sample episodes recorded at -70 mV in ACSF before (upper traces) and 15 min after addition of 100 nM TTX to the bath (lower couple of traces). Mean frequencies and amplitudes were obtained as described in Methods and evaluated with a Mann-Whitney-U-test with confidence levels P values listed in the graphs. Synaptic drives (indicated by asterisks) in sEPSC recordings were excluded from the analysis. C–The neurons were dialyzed with intracellular solution contained Cs+ + TEA (see Methods). Under these conditions the calcium current evoked by depolarization (upper panel) and HCN current activated during hyperpolarization step (lower panel) were clearly isolated. I-V curves for steady state currents are shown on the right. Mean values were obtained from 12 cells patched in slices from RTT and WT and the bars indicate ±SEM. The currents were not normalized to membrane area, because the capacitances in measured cells were around the same. (TIF) [file pone.0202802.s001.tif]

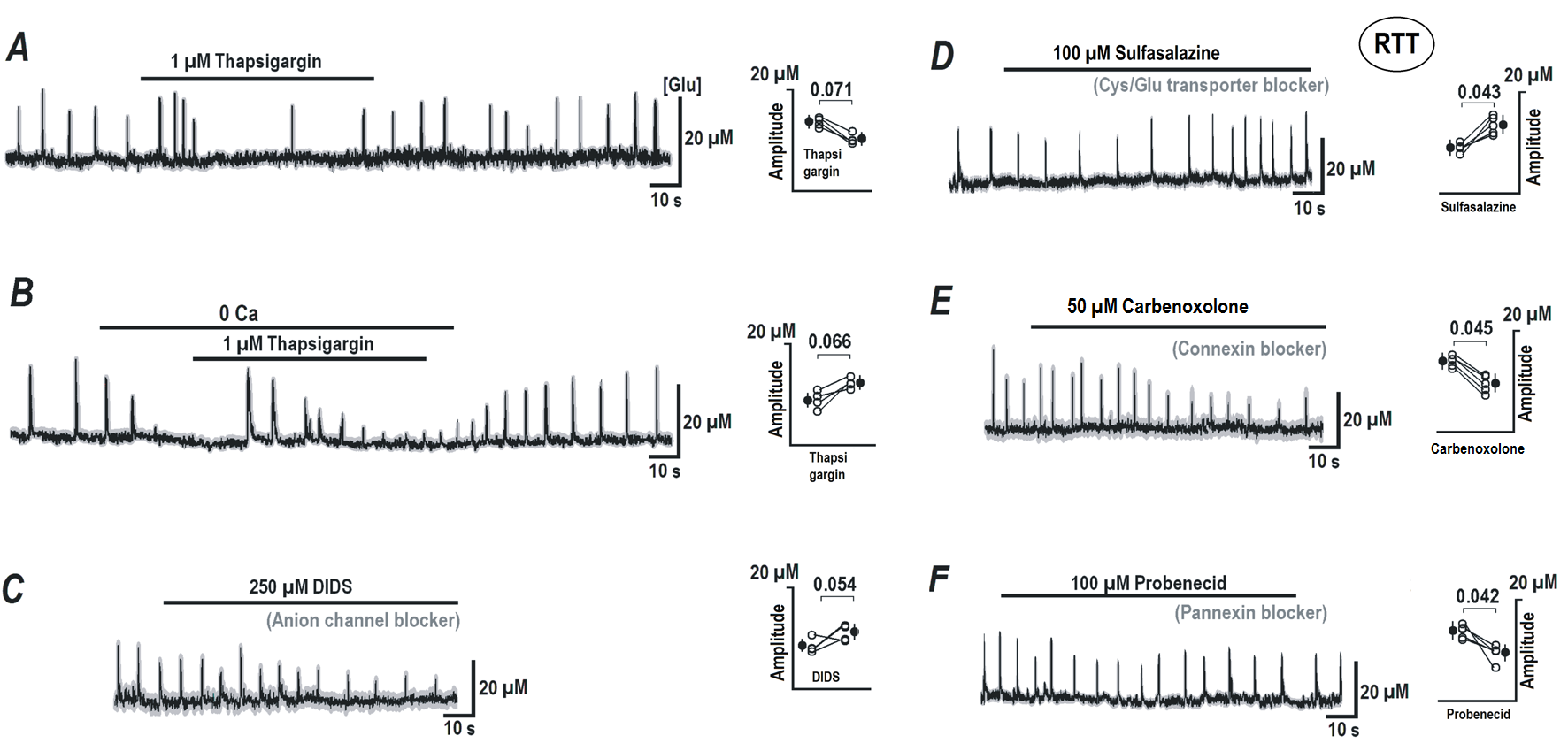

Supplement: S2 Fig — A–Thapsigargin (inhibitor of SERCA that mediates calcium uptake into internal stores) transiently potentiated glutamate spikes in ACSF that was followed by depression. Glutamate spikes recovered to the control values after the drug was washed out. B–Suppression of glutamate spikes in calcium-free solutions was countered by thapsigargin, but the effect was short-lasting. The data in these two experiments indicate dependence of glutamate spikes on intracellular calcium that is transiently released from internal stores after SERCA inhibition by thapsigargin. C—DIDS (4, 4’-Diisothiocyano-2, 2’-stilbenedisulfonic acid, an inhibitor of anion exchange reported to mediate glutamate release from astrocytes) slightly inhibited the amplitude and frequency of glutamate spikes. D–Sulfasalazine, a blocker of Cys/Glu transporter reported to release glutamate from astrocytes, enhanced the amplitude and frequency of glutamate spikes. E, F–Carbenoxolone (a blocker of gap junctions formed by connexins) and probenecid (a blocker of hemi-channels transporting small organic anions) decreased the amplitude and frequency of glutamate spikes. All traces were obtained in CA1 area of RTT animals. The data were evaluated before and after applications of blockers with a Student’s t test. The corresponding P values are listed in group summary. (TIF) [file pone.0202802.s002.tif]

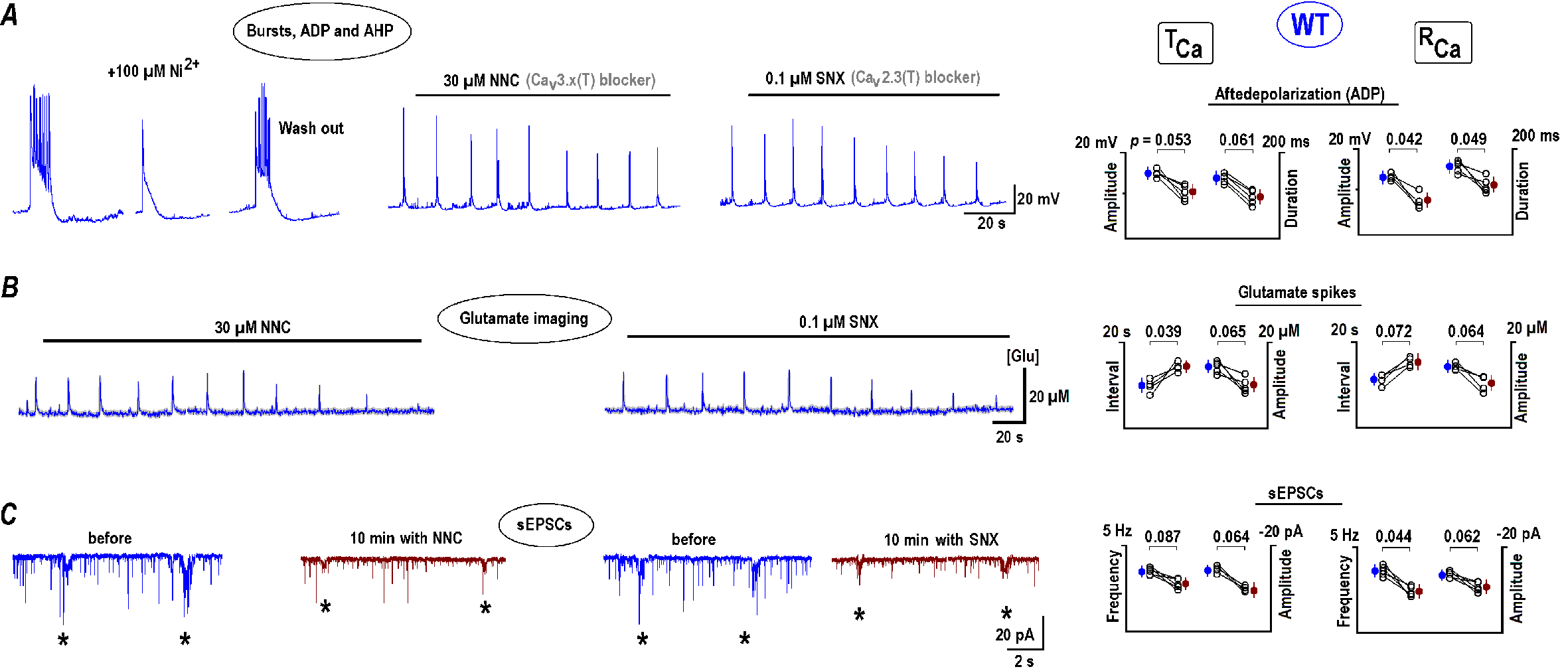

Supplement: S3 Fig — The data were obtained in CA1 neurons from organotypic slices derived from WT animals (the results of experiments in RTT slices are presented in Fig 5 in the main text). A–Current-clamp recordings from CA1 neurons. The traces on the left show inhibition of afterdepolarization (ADP) and bursting activity by Ni2+. The next panels on the right present actions of T- and R-type channel blockers, NNC and SNX. They suppressed ADP and unmasked afterhyperpolarization (AHP). The blockade was accompanied by decreases in the synaptic and bursting activities. B–T- and R-type channel blockers decreased the amplitude and frequency of glutamate spikes. C–Spontaneous EPSCs before and 10 min after application of T- and R-type blockers (the recordings were made in ACSF at the holding potential -70 mV). Both EPSCs and related synaptic drives (indicated by asterisks) were suppressed. Group summary is presented on the right and evaluated with a Student’s t test and corresponding P values are listed in the graphs. (TIF) [file pone.0202802.s003.tif]

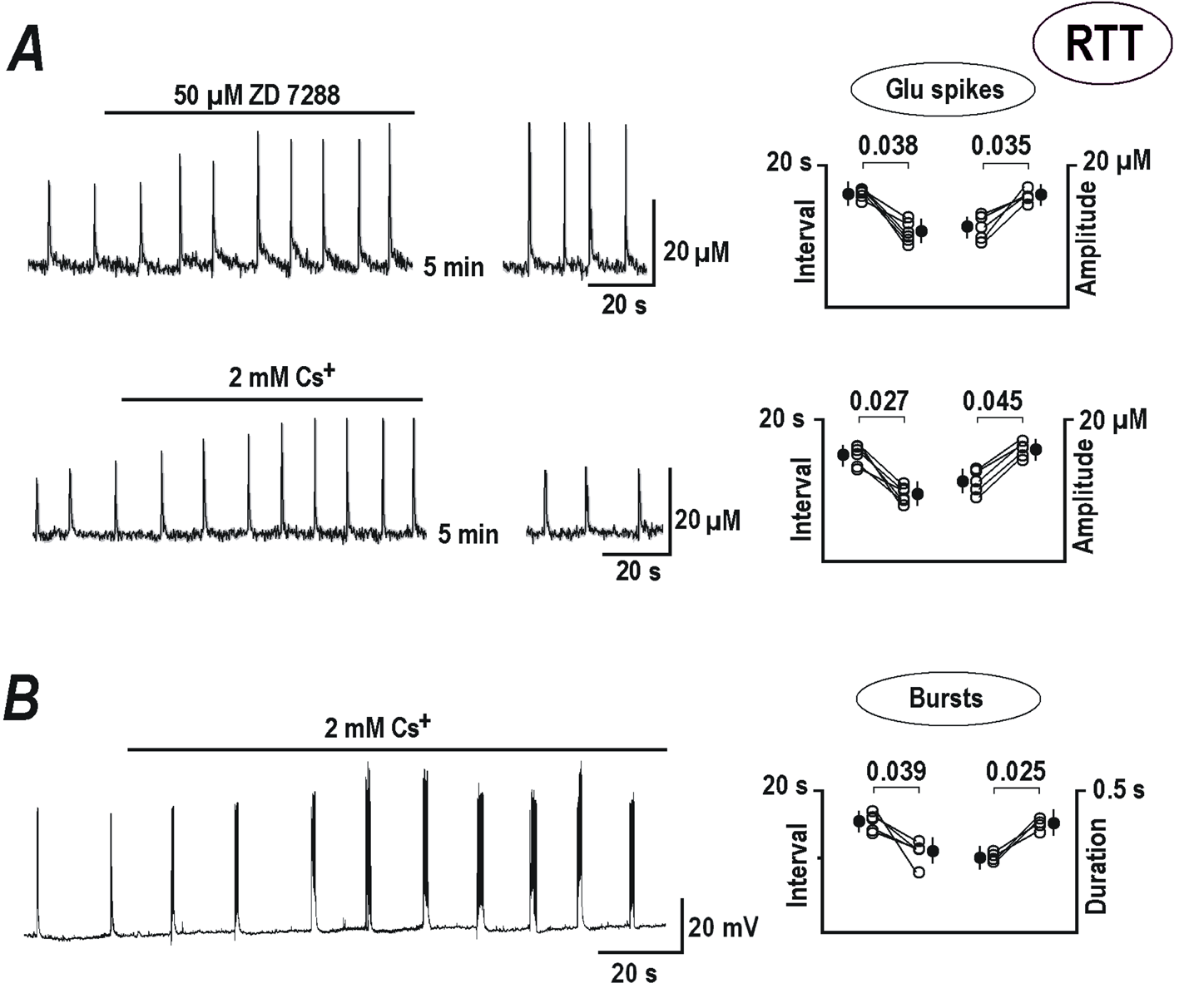

Supplement: S4 Fig — A–HCN blockers augmented the amplitude and frequency of glutamate spikes. ZD 7288 actions were irreversible and did not recover during wash out, whereas Cs+ effects reversed fully after wash out for 5 min. B–The bursting activity was reinforced by Cs+ and increased the duration of bursts and decreased the interval between them. The data were evaluated before and after applications of blockers with a Student’s t test and corresponding P values are listed in group summary. (TIF) [file pone.0202802.s004.tif]
