# Supplementary material for: Regenerative glutamate release in the hippocampus of Rett syndrome model mice
Source: PLoS One. 2018 Sep 26;13(9):e0202802. doi: 10.1371/journal.pone.0202802 (PMC6157837; doi:10.1371/journal.pone.0202802)
Supplement: S1 File — (DOCX) [file pone.0202802.s005.docx]

Experiments on CA1 neurons from age matched acute slices prepared from WT and RTT mice showed similar trends in electrophysiological characteristics to that of organotypic slices. CA1 neurons from RTT neurons showed increased excitability in comparison to WT neurons. On average WT CA1 neurons fired 10.46 ± 1.15 APs in comparison to RTT CA1 neurons, showing 22.04 ± 1.57 APs (n=8, *P*<0.05, Mann-Whitney-U-test) in response to 400 pA (500 ms) current pulse injection.

EPSCs in CA1 neurons showed different features among WT and RTT acute slices. Spontaneous EPSCs (sEPSC) in WT CA1 neurons have a mean frequency of 1.17 ± 0.42 (n=8) in comparison to RTT, which shows an increased mean sEPSC frequency of 1.67 ± 0.38 (n=8, *P*<0.05, Mann-Whitney-U-test). Contrarily, the amplitude of sEPSC in WT and RTT did not show statistically significant difference (12.03 ± 3.18 and 16.94 ± 3.2 pA respectively, n=8, *P*>0.05, Mann-Whitney-U-test).

Miniature EPSCs (mEPSCs) measured in presence of 100 nM TTX to stop spontaneous activities from WT and RTT CA1 neurons in acute slices. Mean mEPSC frequency in WT CA1 neurons was 0.83 ± 0.36 (n=8), whereas; RTT CA1 neurons have significantly higher mean frequency of 1.18 ± 0.35 (n=8) *P*<0.05, Mann-Whitney-U-test). As in the case of sEPSCs, the mEPSC amplitude also did not show significant difference between WT and RTT CA1 neurons (10.01 ± 2.73 and 13.82 ± 3.01 pA respectively, n=8, *P*>0.05, Mann-Whitney-U-test).

Calcium and HCN currents were also measured in response to voltage steps in these neurons and compared. WT CA1 neurons showed on average a calcium current of -19.38 ± 3.21 pA, when stepped to a voltage of -50 mV, whereas RTT CA1 showed significantly higher calcium current of 30.13 ± 3.21 pA at the subthreshold potential of -50 mV. On the contrary, RTT CA1 neurons showed a significantly lower HCN current of 22.41 ± 2.82 pA in comparison to WT CA1 neuron’s amplitude of 76.63 ± 7.34 pA.
