# Supplementary material for: Regenerative glutamate release in the hippocampus of Rett syndrome model mice
Source: PLoS One. 2018 Sep 26;13(9):e0202802. doi: 10.1371/journal.pone.0202802 (PMC6157837; doi:10.1371/journal.pone.0202802)
Supplement: S2 File — (DOCX) [file pone.0202802.s006.docx]

Effects of blockers of additional signaling pathways were tested in order to elucidate their roles in glutamate transient propagation. Thapsigargin (1 µM) is applied to block the SERCA channels and cause the release of stored Ca^2+^ from ER. Thapsigargin reduced the amplitude of glutamate transients from 12.72 ± 1.93 to 10.11 ± 1.82 pA (n=5, *P*>0.05, Student’s t test, S2 Fig. A). Application of thapsigargin in the absence of extracellular Ca^2+^ increased the amplitude of glutamate transients from 9.52 ± 2.73 to 11.88 ± 1.81 pA (n=5, *P*<0.05, Student’s t test). Additionally, application of anion channel blocker (DIDS) did not reduce the mean amplitude of glutamate transients significantly (from 9.73 ± 2.45 to 10.68 ± 2.63 pA, S2 Fig. C, n=4, *P*>0.05, Student’s t test). Further, the involvement of cysteine-glutamate antiporters (SXC) in astrocytes, in generating the glutamate transients seen in RTT slices is tested. Application of 100 µM sulfasalazine, a blocker of SXC, resulted in an increase in the amplitude of glutamate transients (from 9.41 ± 2.34 to 14.07 ± 3.1 µM, S2 Fig. D, n=5, *P*<0.05, Student’s t test). Glutamate release through gap junctions are next tested by treating RTT slices with connexin channel blocker carbenoxolone (50 µM). This treatment significantly reduced the mean amplitude of glutamate transients from 18.52 ± 1.85 to 11.14 ± 2.1 µM (S2 Fig. E, n=5, *P*<0.05, Student’s t test). We also tested the association of spontaneous glutamate release in RTT slices with respect to pannexin channel function. Blockade of pannexin channels with 100 µM probenecid resulted in the reduction of glutamate transient amplitude from 17.64 ± 2.2 to 10.78 ± 2.3 µM, S2 Fig. F (n=4, *P*<0.05, Student’s t test).
