# Supplementary material for: Regenerative glutamate release in the hippocampus of Rett syndrome model mice
Source: PLoS One. 2018 Sep 26;13(9):e0202802. doi: 10.1371/journal.pone.0202802 (PMC6157837; doi:10.1371/journal.pone.0202802)
Supplement: S3 File — (DOCX) [file pone.0202802.s007.docx]

The function of these two classes of calcium channels in the generation and maintenance of spontaneous burst activity and glutamate transients, when present, in WT CA1 neurons is analyzed, as in the case of RTT neurons (Fig. 6). Application of 100 µM Ni^2+^, almost completely abolished the generation of spontaneous bursts in WT CA1 neurons. Upon wash out the previous activity was restored. To test the contribution of T-and R- calcium channels individually, specific drugs against these channels were applied separately. Perfusion of 30 µM NNC 55-0396 (T-type blocker), during induction (100 pA current injection) of regular APs, resulted in the reduction of ADPs and unmasked AHPs. The amplitude of ADPs reduced from 15.21 ± 1.73 to 11.71 ± 2.13 mV, but not significantly, *P*>0.05, Student’s t test, n=4. Additionally the duration of ADP responses also reduced after NNC application (from 132 ± 11.51 to 97.35 ± 10.14 ms, not significant, *P*>0.05, Student’s t test, n=4). Similarly, application of SNX 482 (0.1 µM), also caused a reduction of the amplitude of ADPs (from 12.03 ± 2.1 to 8.94 ± 2.2 mV, n=4, *P*<0.05, Student’s t test) and the duration from 174.17 ± 14.2 to 110.04 ± 13.82 ms (n=4, *P*<0.05, Student’s t test).

Application of NNC to spontaneous glutamate transient showing WT slices, resulted in the reduction of glutamate transients from 13.17 ± 2.6 to 9.53 ± 2.83 µM (n=4, *P*<0.05, Student’s t test), and the interval between glutamate transients increased from 10.13 ± 1.12 to 16. 63 ± 1.21 s, n=4, *P*<0.05, Student’s t test). Similarly, exposure to SNX to block R-type channels to spontaneously active slices reduced the amplitude of glutamate transients from 13.84 ± 2.5 to 11.08 ± 1.8 µM (n=4, *P*>0.05, Student’s t test) and increased the interval from 12.45 ± 3.1 to 17.31 ± 3.4 s (n=4, *P*>0.05, Student’s t test).
