# Supplementary material for: Regenerative glutamate release in the hippocampus of Rett syndrome model mice
Source: PLoS One. 2018 Sep 26;13(9):e0202802. doi: 10.1371/journal.pone.0202802 (PMC6157837; doi:10.1371/journal.pone.0202802)
Supplement: S4 File — (DOCX) [file pone.0202802.s008.docx]

We further tested if blocking HCN channels in hyperexcitable RTT slices with regular glutamate transients would alter their appearance. Inhibition of HCN channels with the specific blocker ZD 7288 resulted in a significant increase in the frequency of glutamate transients (the interval between the spikes decreased from 18.32 ± 2.2 to 10.43 ± 2.4 s, n=5, *P*<0.05, Student’s t test, S4 Fig. A top). ZD 7288 has a stimulating effect on the amplitude of glutamate transients (increase from 11.30 ± 2.4 to 17.64 µM, n=5, *P*<0.05, Student’s t test, S4 Fig. A top).

Cs^+^ is additionally used to block HCN channel conductance in spontaneous glutamate transient displaying slices. The effect of 2 mM Cs^+^ resembled the results with ZD 7288, whereby, the mean interval between spikes decreased from 16.44 ± 2.3 to 9.34 ± 2.4 s (n=5, *P*<0.05, Student’s t test) and the amplitude increased from 11.42 ± 2.6 to 18.03 ± 2.1 µM (n=5, *P*<0.05, Student’s t test, S3 Fig. A bottom). Cs^+^ also altered and increased the AP bursting activity in RTT CA1 neurons. 2mM Cs^+^ caused an elevation in the frequency (interspike interval decreased from 16.93 ± 1.92 to 10.34 ± 2.2 s (n=5, *P*<0.05, Student’s t test, S3 Fig. B) and an increase in the duration of AP bursts from 0.27 ± 0.4 to 0.44 ± 0.03 s (n=5, *P*<0.05, Student’s t test, S3 Fig. B)
